# The Use of Antihypertensive Drugs as Coadjuvant Therapy in Cancer

**DOI:** 10.3389/fonc.2021.660943

**Published:** 2021-05-20

**Authors:** José A. Carlos-Escalante, Marcela de Jesús-Sánchez, Alejandro Rivas-Castro, Pavel S. Pichardo-Rojas, Claudia Arce, Talia Wegman-Ostrosky

**Affiliations:** ^1^ Plan de Estudios Combinados En Medicina (PECEM) (MD/PhD), Facultad de Medicina, Universidad Nacional Autónoma de México, Mexico City, Mexico; ^2^ Facultad de Ciencias Biológicas y Agropecuarias, Universidad Veracruzana, Orizaba-Córdoba, Mexico; ^3^ Escuela Nacional de Ciencias Biológicas, Instituto Politécnico Nacional, Mexico City, Mexico; ^4^ Faculty of Health Sciences, Universidad Autónoma de Baja California, Tijuana, México; ^5^ Medical Oncology/Breast Tumors, Instituto Nacional de Cancerología, Mexico City, Mexico; ^6^ Basic Research Subdirection, Instituto Nacional de Cancerología, Mexico City, Mexico

**Keywords:** cancer, antihypertensive agents, repurposable drugs, Renin – Angiotensin – Aldosterone System, cancer therapy

## Abstract

Cancer is a complex group of diseases that constitute the second largest cause of mortality worldwide. The development of new drugs for treating this disease is a long and costly process, from the discovery of the molecule through testing in phase III clinical trials, a process during which most candidate molecules fail. The use of drugs currently employed for the management of other diseases (drug repurposing) represents an alternative for developing new medical treatments. Repurposing existing drugs is, in principle, cheaper and faster than developing new drugs. Antihypertensive drugs, primarily belonging to the pharmacological categories of angiotensin-converting enzyme inhibitors, angiotensin II receptors, direct aldosterone antagonists, β-blockers and calcium channel blockers, are commonly prescribed and have well-known safety profiles. Additionally, some of these drugs have exhibited pharmacological properties useful for the treatment of cancer, rendering them candidates for drug repurposing. In this review, we examine the preclinical and clinical evidence for utilizing antihypertensive agents in the treatment of cancer.

## Introduction

Cancer is the second leading cause of mortality in individuals younger than 70 years, just after cardiovascular diseases (1). Despite advances in the diagnostic, medical, and interventional fields, the number of new cancer cases increased by 33% from 2005-2015. Recent statistics show that in 2020 19,292,789 new cases were reported and 9,958,133 deaths were caused by cancer worldwide (2), being more relevant because cancer can be present at any age (3).

Cancer is a multifactorial and complex group of diseases that involves dynamic changes in the genome caused by an uncontrolled division of cells with the ability to spread to surrounding tissues (4). These changes are caused by endogenous factors, such as genes, hormones, age, and sex, as well as exogenous factors, such as solvents, ionizing radiation, or drug intake (5–7). In order to provide an organizing framework of cancer amidst all its diversity and complexity, Hanahan and Weinberg proposed the existence of eight principles or hallmark capabilities and two enabling characteristics common to all cancers. These hallmarks of cancer are sustaining proliferative signaling, evading growth suppressors, resisting cell death, inducing angiogenesis, dysregulating energy metabolism, evasion of immune destruction, activating invasion and metastasis, enabling replicative immortality; and the enabling characteristics are genome instability and mutation, and tumor promoting inflammation (8).

Treatment for this complex illness involves radiotherapy, chemotherapy and surgery, which are the most common therapies (9). However, pharmacological treatment continues to develop and to search for more efficient treatments. During the process of drug development, drug repositioning appears to be an important source of possible pharmacological alternatives for cancer treatment.

Drug repurposing is a drug development strategy based on the idea of reusing existing drugs for new medical indications. This strategy has been considered an important alternative in many fields of medicine, especially in complex disorders (10, 11). Repurposing drugs represents an important advantage compared to developing new drugs, not only by economic standards but also by reducing the time to bring a new treatment to patients (12). Currently available drug products are considered a reservoir of agents with the potential to make important contributions in the oncology field (11).

Hypertension is the leading cause of cardiovascular disease and premature death worldwide. Consequently, there are a wide variety of drugs for treating this health issue (13, 14). Hence, repurposing of these drugs could be relevant as adjuvant treatment in cancer because antihypertensive drug targets can also affect the development of malignancy, either directly or indirectly, or both. *In vitro* evidence for the efficacy of antihypertensive drugs in different cell lines showed that they may have a coadjuvant effect against chemoresistant cell lines and may inhibit cell growth and increase chemosensitivity in different types of cancer (15–18). Additionally, these drugs are well tolerated, orally administered, and off-patent, making them cheaper than other cancer treatments (19).

This review aims to explore the repositioning of antihypertensive drugs as an adjuvant therapeutic option in cancer. Other aspects of antihypertensives in the context of cancer, such as the epidemiological association between these drugs and cancer, will not be discussed here. Although carcinogens and cancer chemotherapeutics are substances that share several biological effects, such as DNA damage induction, it should be noted that they are distinguished based on the cellular context: carcinogens select for apoptosis-resistant clones through oncogenic or non-oncogenic processes, whereas anticancer agents are aimed at suppressing cancer cells exploitation of different pathways than the carcinogen that originally selected for them (20).

## Antihypertensive Drugs and Cancer

**Figure 1:**
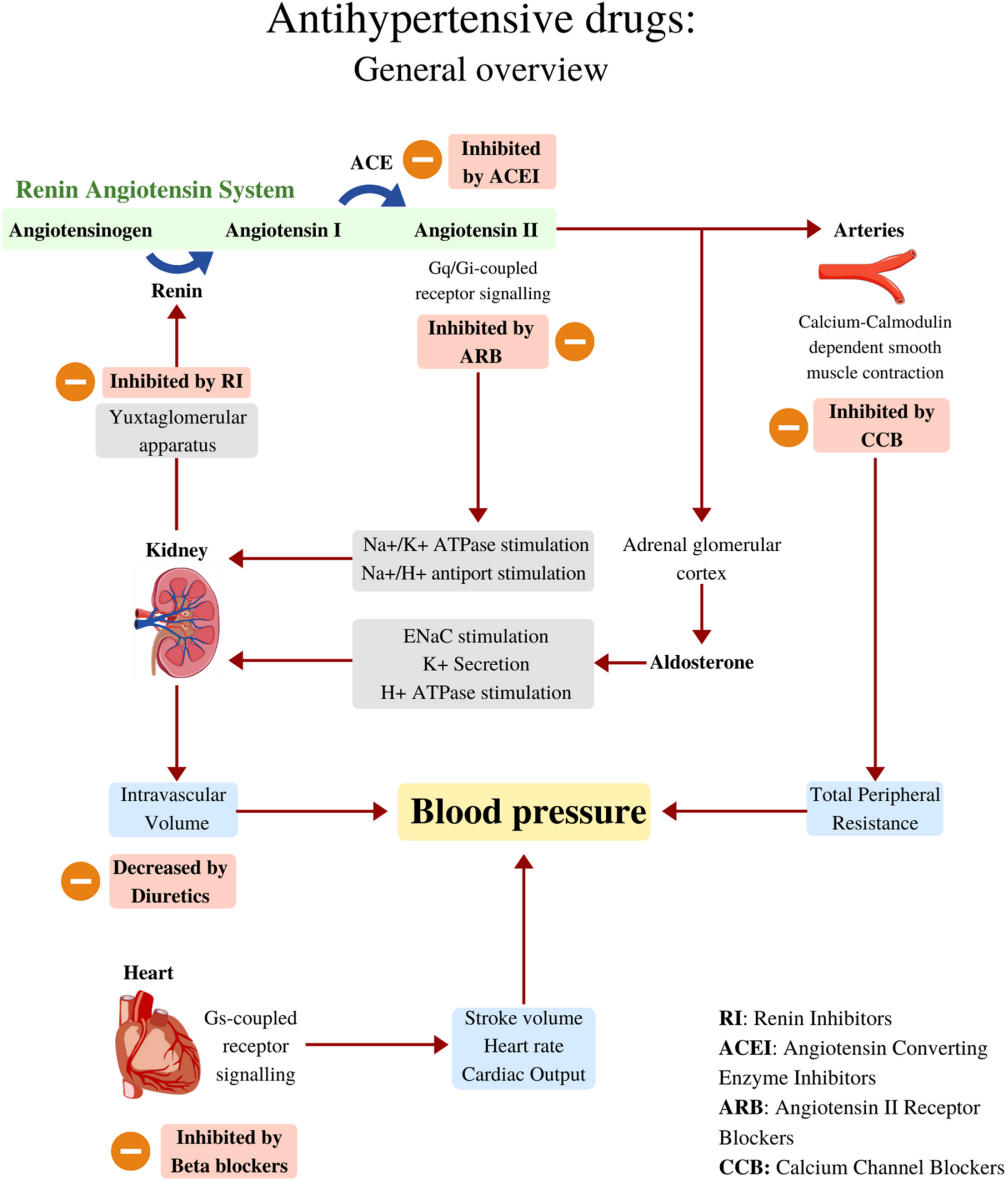
Antihypertensive drugs: General overview. Blood pressure can be determined by changes in cardiac output, total peripheral resistance and intravascular volume. The Renin Angiotensin System is one of the key regulators of blood pressure, it works by increasing Angiotensin II, a powerful systemic vasoconstrictor and one of the main intravascular volume regulators. Angiotensin II works by activating Angiotensin II receptors, which are G-Coupled. Angiotensin II works hand-in-hand with aldosterone to promote sodium and water reabsorption, and hence, maintaining intravascular volume as needed. The heart as a pump, is another blood pressure regulator, it modulates important variables such as Stroke Volume and Heart Rate, which are an important influence for Cardiac Output. Several drugs can lower blood pressure by inhibiting different physiological mechanisms shown in this figure. RI, Renin Inhibitors; ACE, Angiotensin converting enzyme; ACEI, Angiotensin converting enzyme Inhibitors; CCB, Calcium-Channel Blockers.

Antihypertensive drugs can be classified into four main groups according to their mechanism of action: those that act in the renin angiotensin aldosterone system (RAAS), either by inhibiting angiotensin converting enzyme (ACE), blocking the angiotensin type 1 receptor (AT1R), directly inhibiting renin action, or by antagonizing aldosterone binding to its receptor; those that act blocking the calcium channels, which can block either dihydropyridine or non-dihydropyridine calcium channels; beta blockers that block the β-adrenergic receptors; and diuretics, which decrease the volume in the circulatory system (21). These mechanisms are summarized in **Figure 1**.

The role antihypertensive drugs may play in cancer treatment remains unclear, considering that there are reports showing that some antihypertensives increase the risk of developing several neoplasms (22, 23). This does not automatically preclude antihypertensive drugs from being useful as adjuvants for cancer treatment. For instance, several known carcinogens, such as arsenic, tamoxifen or phorbol ester, are also effective treatments for other cancers (20). In the case of antihypertensive drugs, for instance, calcium channel blockers (CCBs) are associated with intracellular calcium accumulation, which promotes apoptosis and makes them potentially useful for the treatment of cancer, even if short-release CCBs have been associated with cancer (24–27).

**Figure 2:**
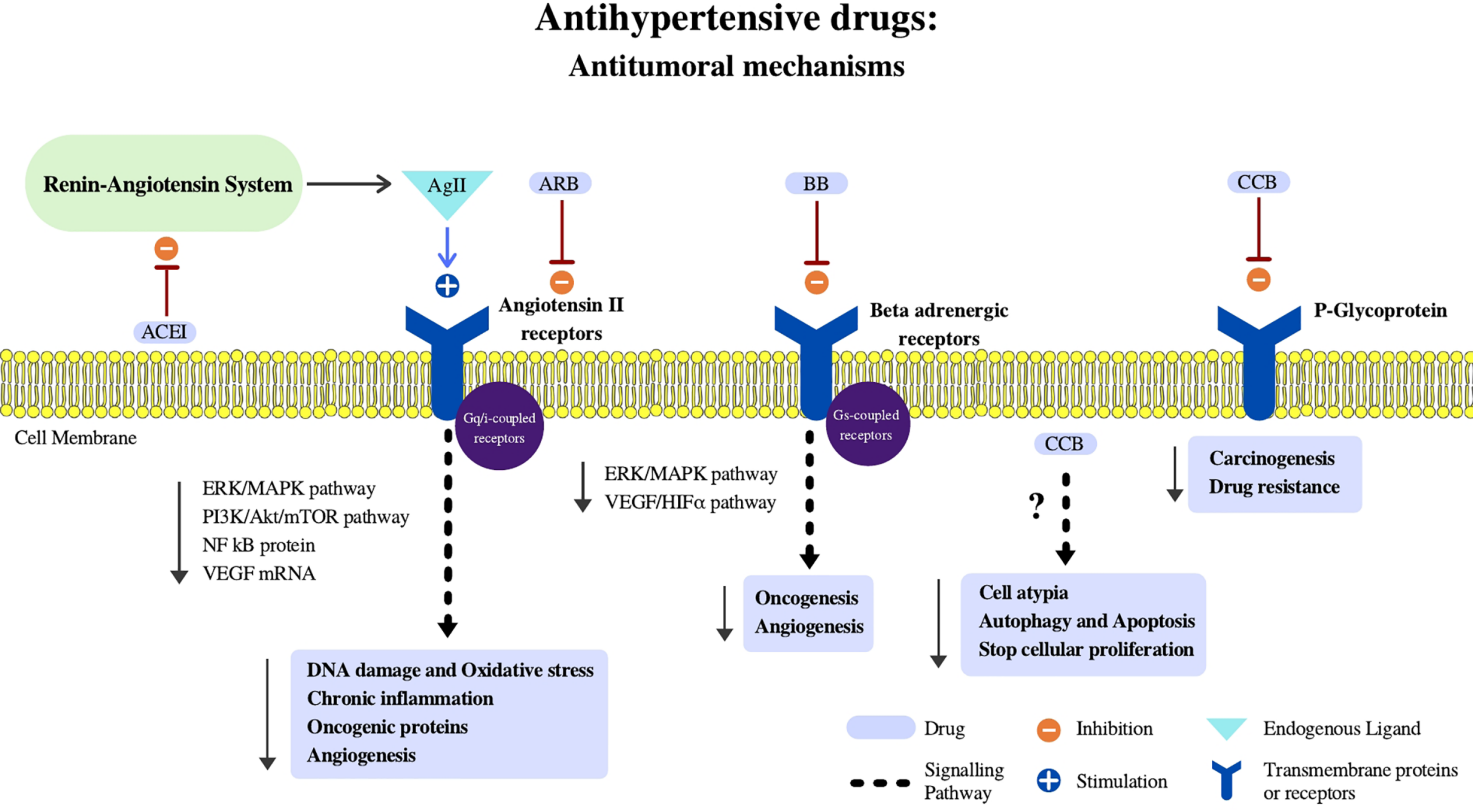
Antihypertensive drugs: Antitumoral mechanisms. In this figure we summarize potential mechanisms in which antihypertensive drugs may aid oncologic therapies throughout different cellular effects schematized in the figure. Ag II, Angiotensin II; Epi, Epinephrine; NFkB, Nuclear factor kappa B; VEGF, Vascular Endothelial Growth Factor; HIFα, Hipoxia Induced Factor Alpha; ARB, Angiotensin II Receptor Blockers; BB, Beta Blockers; CCB, Calcium Channel Blockers.

Considering *in vitro, in vivo* and clinical evidence, four principal antihypertensive groups of drugs as cancer adjuvants will be discussed below. The cellular mechanisms in which antihypertensives exert their effects in cancer cells are described in **Figure 2** and will be approached in the context of the hallmarks of cancer in **Table 1**. Additionally, we conducted a review at clinicaltrials.gov looking for studies from July 15th to March 8th of this year, that had the objective of repositioning antihypertensive drugs as adjuvant therapy in cancer were selected. The keywords used in the search were “cancer” as a condition, and the other terms were candesartan, captopril, diltiazem, enalapril, lisinopril, losartan, nicardipine, nifedipine, ramipril, telmisartan, valsartan, verapamil, delapril, fosinopril, cilazapril, spirapril, imidapril, quinapril, irbesartan, and felodipine. This search yielded 10 non duplicated trials, that are detailed in **Table 2**.

**Table 1:** Hallmarks of cancer affected by antihypertensive drugs alone or in synergy with other drug in preclinical and clinical studies.

Hallmark of cancer	Drug category	Antihypertensive Drugs	Mechanism	Sinergism	Source
Resisting cell death	Aldosterone antagonist	Spironolactone	-Reduces survivin mRNA expression	NA	(28)
			-Increases protein degradation by proteasomes.		
	β-blocker	Propranolol	-Downregulation of Bcl-2	NA	(29)
			-Upregulation of Bax and other pro-apoptotic molecules		
	CCB	Mibefradil^a^	-Inhibition of T-type VGCC results in cell death mediated by BAX and p27.	Temozolomide	(30, 31)
		Verapamil	-Apoptosis in a myeloma cell line through unfolded protein response and Jun N-terminal kinase activation	Bortezomib	
			- Autophagy-like process in prostate cancer and colon adenocarcinoma cell lines.		
		Diltiazem	-Reduced interaction between bak and Bcl-xL	Bortezomib	
	CBB	Amlodipine	-Promotes Ca^2+^ entry, inhibiting YAP/TAZ signaling.	NA	(32)
Deregulating cellular energetics	β-blocker	Atenolol	-Inhibition of respiratory chain breast cancer cell lines, thus, reducing oxygen consumption.	Metformin	(33)
		Propranolol	-Inhibition of hexokinase 2 and GLUT1 transporter.	Vemurafenib	(34)
Sustaining proliferative signaling	ACEI	Captopril, trandolapril	-Increase apoptosis correlated with reduced expression of *MYC.*	NA	(35)
	ARB	Candesartan	-Active metabolites of candesartan inhibit EGF signaling.	NA	(36)
		Losartan	-Inhibition of the growth factors bFGF and PDGF.	NA	(37–39)
			-Inhibits PI3K/AKT pathway		
	β-blocker	Non-selective β-blockers (propranolol, carvedilol)	-Co-inhibition of EGFR signaling and JNK/SAPK pathway	Afatinib	(40, 41)
	CCB	Amlodipine	-Reduces the phosphorylation of EGFR	NA	(42) (32)
			-Promotes Ca^2+^ entry, inhibiting YAP/TAZ signaling.		
		Verapamil	-Reduced *EGFR* mRNA expression, impairing EGF signaling.	NA	(43)
Evading growth suppressors	ACEI	Perindopril, fosinopril	-Downregulation of cyclin D1, arresting cell cycle at G1.	NA	(44)
	ARB	Losartan	-Inhibits production of cyclin D1, preventing progression across the G1 phase of the cell cycle.	NA	(44)
	β-blocker	Propranolol	-Increases the fraction of cells in G0/G1	NA	(29)
	CCB	Amlodipne, nicardipine	-Reduced intracellular Ca^2+^ concentration inhibits several proteins necessary for cell cycle progression.	NA	(45)
			-Increases the negative cell cycle regulator p21^Waf1/Cip1^.		
Avoiding immune destruction	ACEI	Captopril	-Hypersegmentation and induction of cytotoxic activity of tumor-associated neutrophils, mediated by mTOR.	NA	(46, 47)
			-Increases antitumor T cells and reduces immunosuppressive cells.		
	ARB	Valsartan, Candesartan	-Upregulation of antitumoral T cells (CD3^+^ and CD8^+^) and reduction of immunosuppressive cells activity.	Anti-PD-L1 and anti-CTLA4 antibodies.	(48)
	Aldosterone antagonist	Spironolactone	-Increased surface expression of NKG2DL, recognized by NK cells. This is mediated by RXRγ rather than the MR.	NA	(49)
	β-blocker	Propranolol	-Inhibition of adrenergic signaling upregulates tumor-infiltrating CD8^+^ T cells.	NA	(50)
Activating invasion and metastasis	ACEI	Captopril, perindopril, fosinopril	-Direct inhibition of matrix metalloproteinase activity	NA	(44, 51)
			- Fosinopril decrease TFG-β activity.		
	ARB	Losartan	-Downregulation of TFG-β	FOLFIRINOX	(44, 52)
	Aldosterone antagonist	Spironolactone	-Activation of RXRγ, which promotes the expression of antimetastatic gens *TIMP2* and *TIMP3*.	NA	(49)
	β-blocker	Propranolol	-Inhibition of stress-induced metastasis, mediated by M2 macrophages. -Downregulation of MMP-2 and MMP9	NA	(53, 54)
	CCB	Cilnidipine, manidipine, felodipine, amlodipine	-Inhibition of filopodia formation and stability, regulated by an L-type VGCC.	NA	(55)
Inducing angiogenesis	ACEI	Perindopril, benazepril, captopril	-Downregulation of *VEGF* transcription.	Intereferon α, Cimetidine, meloxicam	(56, 57)
	ARB	Candesartan, losartan, Olmesartan	-Downregulation of *VEGF* transcription.	Gemcitabine Sorafenib	(56, 58, 59)
			Inhibition of IGF-I		
	β-blocker	Propranolol	-Inhibition of tubulogenesis of endothelial cells and MMP-9 secretion. reduces the mRNA expression of *VEGF*.	NA	(54)

^a^Mibefradil is no longer used as antihypertensive drug due to its conflicting drug interaction profile.

ACEI, Angiotensin converting enzyme inhibitor; ARB, angiotensin receptor blocker; bFGF, basic fibroblast growth factor; CCB, calcium channel blocker; EGF, epidermal growth factor; EGFR, epidermic growth factor receptor; FOLFIRINOX, folinic acid, 5-fluoruacil, irinotecan, oxaliplatin; IGF-I, insulin-like growth factor I; MMP, matrix metalloproteinase; MR, mineralocorticoid receptor; mTOR, mechanistic target of rapamycin; NK, natural killer; NKG2DL, natural killer group 2D receptor ligand; PDGF, platelet-derived growth factor; RXRγ, retinoid X receptor gamma; TFG-β, transforming growth factor β; VEGF, vascular endothelial growth factor; VGCC, voltage-gated calcium channel.

**Table 2:** Clinical trials found in clinicaltrials.gov studying antihypertensive drugs in cancer.

Title	Condition	Interventions	Phase	Location	Status	Purpose
*In Vivo* Angiotensin Generation Using Tissue Plasminogen Activator and Captopril in Treating Patients with Progressive Metastatic Cancer	-Unspecified adult solid tumor	-Recombinant tissue plasminogen activator	Phase 1	USA	Completed	This trial studied the side effects and best dose of tissue plasminogen activator and captopril and saw how well they treated patients with progressive metastatic cancer.
		-Captopril	Phase 2			
A Proof-of-concept Clinical Trial Assessing the Safety of the Coordinated Undermining of Survival Paths by 9 Repurposed Drugs Combined with Metronomic Temozolomide (CUSP9v3 Treatment Protocol) for Recurrent Glioblastomas	-Glioblastoma	-Temozolomide	Phase 1	Germany	Active, not recruiting	A proof-of-concept clinical trial assessing the safety of the coordinated undermining of survival paths by 9 repurposed drugs combined with metronomic temozolomide (CUSP9v3 treatment protocol) for recurrent Glioblastoma
		-Aprepitant	Phase 2			
		-Minocycline				
		-Disulfiram				
		-Celecoxib				
		-Sertraline				
		-Captopril				
		-Itraconazole				
		-Ritonavir				
		-Auranofin				
Enalapril Maleate and Doxorubicin Hydrochloride in Treating Women with Breast Cancer	-Breast Cancer	-Doxorubicin hydrochloride	Not applicable	USA	Completed	This randomized clinical trial studied enalapril maleate administration together with doxorubicin hydrochloride to see how well it works in treating women with breast cancer.
		-Enalapril maleate			Has results	
Losartan and Nivolumab in Combination With FOLFIRINOX and SBRT in Localized Pancreatic Cancer	-Pancreatic cancer	-FOLFIRINOX	Phase 2	USA	Recruiting	This research study is studying a combination of interventions as a possible treatment for pancreatic tumors.
		-Losartan				
		-Nivolumab				
		-Radiation SBRT				
		-Surgery				
Proton w/FOLFIRINOX-Losartan for Pancreatic Cancer	-Pancreatic Cancer	-FOLFIRINOX	Phase 2	USA	Active, not recruiting	In this research study, they seek to determine whether combining FOLFIRINOX with Losartan before proton radiation therapy will be more efficient at controlling the growth or shrinking of tumors than just FOLFIRINOX alone.
		-Losartan				
		-Proton Beam Radiation			Has results	
Losartan and Hypofractionated Rx After Chemo for Tx of Borderline Resectable or Locally Advanced Unresectable Pancreatic Cancer (SHAPER)	-Pancreatic Cancer	-Losartan	Phase 1	USA	Recruiting	This trial studies the side effects of Losartan and hypofractionated radiation therapy after chemotherapy in treating patients with pancreatic cancer that may or may not be removed by surgery (borderline resectable) or has spread from its original site of growth to nearby tissues or lymph nodes and is not amenable to surgical resection (locally advanced unresectable).
		-Losartan Potassium				
		-Hypofractionated				
		-Radiation Therapy				
Tissue Pharmacokinetics pf intraoperative Gemcitabine in Resectable Adenocarcinoma of the Pancreas	Pancreatic Cancer	-Gemcitabine	Early Phase 1	USA	Terminated	This clinical research studied whether gemcitabine can enter pancreas cancer cells, measure its amount that may enter the cells, and biomarker testing.
		-Losartan				
Imaging Perfusion Restrictions From Extracellular Solid Stress – An Open-label Losartan Study	-Glioblastoma	-Losartan	Phase 2	Norway	Recruiting	To assess Losartan’s dose-response relationship on imaging-based measures of tissue perfusion and mechanical forces in patients with brain tumors.
	-Brain Metastases					
Losartan + Sunitinib in Treatment of Osteosarcoma	-Osteosarcoma	-Losartan	Phase 1	USA	Recruiting	To determine the Maximally Tolerated Dose of Losartan and Sunitinib Combination Therapy.
		-Sunitinib				
Serial Measurements of Molecular and Architectural Responses to Therapy (SMMART) PRIME Trial	-Accelerated Phase Chronic Myelogenous Leukemia, BCR-ABL1 Positive	-Abemaciclib	Phase 1	USA	Recruiting	To determine if samples from a patient’s cancer can be tested to find combinations of drugs that provide clinical benefit for the kind of cancer the patient has. This study tries to understand why cancer drugs can stop working and how different cancers in different people respond to different therapy types.
	-Anatomic Stage IV Breast Cancer AJCC v8	-Abiraterone				
	-Anemia	-Afatinib				
	-Ann Arbor Stage III Hodgkin Lymphoma	-Bevacizumab				
	-Ann Arbor Stage III Non-Hodgkin Lymphoma	-Bicalutamide				
	-Ann Arbor Stage IIIA Hodgkin Lymphoma	-Bortezomib				
	-Ann Arbor Stage IIIB Hodgkin Lymphoma	-Cabazitaxel				
	-Ann Arbor Stage IV Hodgkin Lymphoma	-Cabozantinib				
	-Ann Arbor Stage IV Non-Hodgkin Lymphoma	-Capecitabine				
	-and 50 more	-and 44 more (including losartan) Procedure:-Biospecimen Collection				
Combination of Hydroxyurea and Verapamil for Refractory Meningiomas NCT00706810	-Cancer	-Hydroxyurea	Phase 2	USA	Completed	All subjects underwent images studies to assess tumor measurements within three to four weeks before beginning treatment.
	-Brain Cancer	-Verapamil				
	-Meningioma					
Brentuximab Vedotin, Cyclosporine, and Verapamil Hydrochloride in Treating Patients With Relapsed or Refractory Hodgkin Lymphoma	-Recurrent Hodgkin Lymphoma	-Brentuximab Vedotin	Phase 2	USA	Recruiting	This trial studies the side effects and best dose of brentuximab vedotin and cyclosporine when given together with verapamil hydrochloride in treating patients with Hodgkin lymphoma that has come back (relapsed) or does not respond to treatment (refractory).
	-Refractory Hodgkin Lymphoma	-Cyclosporine				
		-Verapamil				
		-Verapamil Hydrochloride				

This table lists recently completed studies as well as trials that have not been completed or that have not published their results as they appear in https://clinicaltrials.gov/. The conclusion of studies with published results can be consulted in the main text.

## Renin-Angiotensin System-Based Drugs

### Renin-Angiotensin-Aldosterone System

The understanding of cancer development is related to a contemporary perspective of several systems, including the RAAS, a physiological regulator of systemic arterial pressure. However, the current perspective regarding this system is more complicated. It involves a balance between the processing pathways for angiotensin II (Ang II) peptide precursors and its interactions with several receptors that lead in several instances to opposite effects. In addition local activity of several RAAS components independent of systemic RAAS have been observed in different tissues and organs (18).

Intracellular effects of the RAAS system involve the participation of derivatives of angiotensinogen (Ang II and other peptides), principally mediated by AT1R, angiotensin II receptor type 2, MAS receptor, insulin-regulated aminopeptidase receptor, and angiotensin II receptor type 4. Dysregulation of the components of this system has been described in several cancer (e. g., breast, ovary, prostate, pancreas, and gut) and, in some instances, has been correlated with prognosis (18). Signaling through AT1R increases cell proliferation in malignancy in two ways, by directly affecting tumor cells and by modulating vascular cell growth during angiogenesis (8, 18). Growing evidence suggests that Ang II, the main effector of the RAAS, contributes to each sequential step of cancer metastasis by promoting cancer cell adhesion to endothelial cells, transendothelial migration and tumor cell migration across the extracellular matrix (56).

Proposal of RAAS as an active element in cancer is associated with the development of hallmarks of cancer, such as constant angiogenesis, evasion of apoptosis, self-sufficiency in growth signals, tissue invasion and metastases, and limitless replicative potential. This system participates in regulating these capabilities, and many of the increased metastasis and invasion characteristics associated with RAAS expression are likely to be a direct consequence of angiogenesis (18, 56). RAAS affects multiple aspects of cancer, and blocking RAAS has been associated with an improved prognosis in some cancer types.

### Angiotensin-Converting Enzyme Inhibitors (ACEI)

#### Mechanism of Action

In 1981, captopril became the first ACEI drug available and has since been widely used for treating diverse cardiovascular diseases. ACEI drugs act on the RAAS system by inhibiting the formation of Ang II, preventing the downstream effects mediated by AT1R (60, 61). Furthermore, ACEIs do not interfere with the conversion of angiotensin-I to angiotensin-1-9 because they are converted by endopeptidases. After conversion to angiotensin-1-9, it is cleaved by ACE-2, which seems unaffected by classical ACEI, to become angiotensin-1-7, which binds to MAS receptors, causing the opposite effect of AT1R (vasodilatation, apoptosis, antiproliferation) (62–66). Currently, there are many ACEIs in existence in addition to captopril: enalapril, benazepril, and fosinopril and others.

#### Evidence From Studies *In Vitro* and *In Vivo*

There is *in vitro* and *in vivo* evidence for the efficacy of this kind of drugs in cancer treatment. An example of this evidence has been shown using azoxymethane in 45 male C57BL/KsJ-db/db mice to induce premalignant lesions with the aim of comparing the effects of two different drugs on these groups. The results showed that captopril reduced the number of malignant preneoplastic lesions and the amount of DNA damage in the colon, showing that there is an important relationship between ACE activityand cancer, considering that captopril is a RAAS inhibitor and may nullify some of the oncogenic effects of azomethane by attenuating chronic inflammation and reducing oxidative stress (67). Another example comes from a model of pancreatic cancer of transgenic mice randomly treated with placebo, aspirin or enalapril. In this study, enalapril significatively delayed the progression of pancreatic cancer precursor lesions by downregulating NF-kB in tumor cells (68).

Another ACEI perindopril significantly inhibits tumor development and angiogenesis, possibly independently of AT1R blockade, and this inhibitory effect was accompanied by suppression of the vascular endothelial growth factor (VEGF) (69). Other *in vivo* studies have shown that perindopril has a potential inhibitory effect on tumor growth due to suppression of VEGF-induced angiogenesis in head and neck squamous cell carcinoma and renal cell carcinoma (70). Furthermore, it is been shown that captopril can reduce metastatic potential to lungs (70). The efficacy of ACEIs has been attributed to diminished expression of VEGF through AT1R decreasing signaling, in which ERK1/2 and Akt pathways take part downstream (71). ACEIs have been mostly studied in the context of the inducing angiogenesis hallmark, however, they can impair other hallmarks as well, such as evading growth suppressors, avoiding immune destruction and activating invasion and metastasis. A more detailed description of the hallmarks affected can be seen in **Table 1**.

#### Evidence From Clinical Studies

A systematic review suggested that ACEI or ARB use may be associated with improved outcomes in patients with cancer, such as non-small cell lung cancer, pancreatic cancer, or breast cancer; however, a sub-analysis for specific drug classes was not performed (72). A posterior meta-analysis also observed a survival benefit for urinary tract, colorectal and prostate cancer, but it also lacked data for ACEIs alone (73). In patients with pancreatic ductal adenocarcinoma, lisinopril extends the overall survival time independently of chemotherapy. Furthermore, it has been suggested that ACEIs may reduce the malignant potential of cancer cells and stimulate the immune microenvironment in patients with pancreatic ductal adenocarcinoma (74). Other retrospective reports analyzing long-term medication with ARBs and ACEIs in addition to platinum-based first-line chemotherapy suggest that when used in combination, they prolonged survival in patients with advanced lung cancer may result (75). Similar scenarios were found in a phase II trial reporting favorable overall survival outcomes when combining cimetidine, a cyclooxygenase-2 inhibitor and a renin-angiotensin-system inhibitor in metastatic renal cell carcinoma (57). ACEIs may be relevant in hepatocellular carcinoma as well. A systematic review including 3 interventional studies reported that ACEIs taken together with vitamin K or branched-chain amino acids reduced the risk of recurrence of this cancer (76). These data suggest that ACEIs may represent potential adjuvant therapies. Additionally, enalapril was observed to be well tolerated in women with female cancer and it did not alter doxorubicin pharmacokinetics, which open the door for further studies of this combination (77). There are two clinical trials undergoing or recently concluded involving captopril, and study of enalapril in combination with doxorubicin (**Table 2**).

ACEIs are generally regarded as safe and the adverse effects associated to its consumption are well tolerated by most patients. The most common adverse effect reported is dry cough, whereas hyperkalemia and hypotension are also reported. Angioedema is a far rarer event, however, in some cases it could lead to life-threatening scenarios (61). ACEIs may also exacerbate the nephrotoxic effects of certain antineoplastics agents (e.g., cisplatin) (78).

### Direct Renin-Inhibitors

Although ACEIs and ARBs are the most common antihypertensive drugs, RAAS blockers do not guarantee total inhibition of the RAAS. Aliskiren was the first direct renin inhibitor suitable for oral administration.

#### Mechanism of Action

Aliskiren acts by blocking the interaction of circulating renin with angiotensinogen, which leads to angiotensin I (Ang I) formation. As a result, the concentrations of Ang I and its derivative, Ang II, are decreased. In addition to the systemic reduction in blood pressure and vascular resistance, aliskiren also reduce plasma renin activity (79). They are one of the safest antihypertensive drugs because they are not metabolized by cytochrome p450. However, in contrast with the use of ACEIs and ARBs, Aliskiren have demonstrated a negative impact in patients with nephropathy or diabetes. The increase in plasmatic prorenin secondary to aliskiren intake is directly associated with the increase in microalbuminuria (79).

#### Evidence From Studies *In Vitro* and in Animal Models

Even though DRIs have not been investigated as antitumor drugs, their carcinogenic potential in rat studies has been described in a RAASH2 mouse study submitted to the FDA for product registration. Aliskiren may be an unlikely direct therapeutic candidate for cancer, and it may help with comorbidities associated with cancer, such as cachexia. Research suggests that aliskiren delays cachexia development by reducing tumor burden and prolonging survival in mouse models (80).

#### Evidence From Clinical Studies

No clinical studies evaluating the impact of renin-inhibitors on the prognosis of patients with cancer has been published as for 2021.

### Angiotensin-Receptor Blockers (ARBs)

#### Mechanism of Action

ARBs function by blocking AT1R, preventing the binding of Ang II with this receptor (81). This specific blockade by ARBs reduces adverse effects secondary to kinins and substance P accumulation (degraded by ACE under physiological conditions) like cough and angioedema, more frequent in patients receiving ACEIs (82).

#### Evidence From Studies *In Vitro* and in Animal Models

In a study from 2017, human prostate cancer cell lines PC3, DU145, and LNCap-Ln3, growth, cell viability, proliferation and migration were evaluated under the effect of ARBs (fimasartan, losartan, eprosartan and valsartan) at concentrations of 100, 200 and 400 µM. The results showed that ARBs reduced cell viability compared to the control group, and at a concentration of 400 µM, all ARBs exerted antiproliferative effects on prostate cancer cells at each time point examined. Nevertheless, fimasartan exhibited the greatest cytotoxicity, while valsartan demonstrated the lowest antiproliferative activity compared to other ARBs in prostate cancer cells (83). In the same year, telmisartan was showed to inhibit cell proliferation and to induce G_0_/G_1_ arrest in two cholangiocarcinoma cell lines (84). In another study, telmisartan was reported to inhibit proliferation and tumor growth of esophageal squamous cell carcinoma cell lines, also by cell cycle arrest (85). Moreover, telmisartan appears to downregulate Bcl-2, an anti-apoptotic molecule, and to activate caspase-3, thus, inducing cell death, as observed in a cell line derived from human renal cell carcinoma (86). In general, in a similar manner to ACEIs, the main hallmark of cancer addressed by ARBs is angiogenesis, nonetheless, ARBs can interfere with several others (**Table 1**). Moreover, losartan is capable of suppressing YAP signaling, an effector of the Hippo pathway (37). Several hallmarks of cancers, such as resisting cell death, sustaining proliferative signaling, activating invasion and metastasis and avoiding immune destruction have been associated with dysregulating signaling at the Hippo pathway (87).

ARBs can modify tumor desmoplasia (fibrosis of the tumoral stroma) by regulating tumor-associated fibroblasts. Desmoplasia compresses vasculature and impedes infiltration of immune cells. Thereof, alleviation of tumor desmoplasia allows for T cell infiltration and improves perfusion, which in turn increases drug delivery to the site (56).

#### Evidence from Clinical Studies

The CHARM study, evaluating mortality in patients with chronic heart failure receiving candesartan in a double-blind, placebo-controlled manner published in 2004, showed significantly greater cancer mortality with the use of candesartan (88); however, this finding was considered coincidental by the authors after assessment of previous trials including candesartan. The results of this trial and another 12 clinical trials comparing telmisartan, irbesartan, valsartan, candesartan and losartan were condensed in a meta-analysis from 2020, revealing no difference in cancer mortality between patients taking ARBs and controls (89). In 2017, a meta-analysis evaluating 11 studies showed a significantly improved overall survival in patients taking any ARB (73). Concerning site-specific cancers, in an observational study including 878 patients, patients taking ARBs exhibited an improved progression-free survival (90). Improvement in overall survival was also observed for individuals with ovarian cancer taking losartan in a recent retrospective study (91). Clinical trials specifically evaluating the impact of ABRs in ovarian cancer are lacking.

For prostate cancer, a pilot study including 23 patients with hormone-refractory prostate cancer receiving candesartan dating from 2005 reported a decrease in serum levels of prostate-specific antigen in 8 patients and stable or improved performance status (92). However, no other clinical trial was completed thereafter. More data for the potential utility of ARBs came from a nationwide cohort study from Finland, in which ARBs significantly decreased the risk of death after radical prostatectomy, as well as the risk of starting anti-androgen deprivation therapy compared to no use of ARBs (93). The results of this study were confirmed in a larger cohort in Finland including patients in several stages of the disease (94). Even if there exists evidence from observational studies, clinical trials are still necessary to understand the benefits of ARBs in prostate cancer.

Since 2010, RAAS blockade was observed to favorably impact pancreatic cancer mortality, which prompted a phase I clinical assessment of the combination of candesartan and gemcitabine, which was deemed safe for a phase II clinical trial by the same group (95, 96). In a phase II trial including 35 patients with advanced pancreatic cancer, patients receiving 16 mg of losartan had a modest but significant increase in progression-free survival compared to patients taking 8 mg (4.6 vs 3.5 months) (58). In another single-arm phase II clinical trial, in patients with locally advanced pancreatic ductal adenocarcinoma, losartan in combination with a cocktail of several adjuvant chemotherapeutics (FOLFIRINOX) and posterior chemoradiotherapy was useful in achieving complete surgical resection (52). Even if the study comprised a single arm, a parallel phase II trial evaluating FOLFIRINOX without losartan followed by chemoradiotherapy observed a similar rate of complete resection in patients with borderline resectable pancreatic adenocarcinoma, expected to higher by definition than the rate of complete resection of locally advanced tumors, a finding suggesting that losartan may increase their resectability (97). Double-blind, placebo-controlled trials are needed to adequately evaluate the role of losartan in these tumors. 3 other phase I/II trials involving losartan in patients with pancreatic cancer are active on recruiting phase (**Table 2**).

ARBs have been suggested to be useful for treating peritumoral edema, an important cause of impairment in patients with glioblastoma. A cohort study observed that individuals taking RAS blockers, primarily ARBs, required a significantly reduced dose of steroids, the drug of choice for treating peritumoral edema (98). A cross-sectional study in patients with glioblastoma added to the evidence, showing that intake of ARBs was significantly associated with reduced edema volume as measured by magnetic resonance (99). However, a multicenter, double-blind, placebo-controlled trial assessing the addition of losartan to standard treatment for glioblastoma (ASTER trial) in 75 patients (1:1 arms ratio) found no difference in the dosage of steroids prescribed between the groups (100). A losartan phase II trial in patients with glioblastoma is currently recruiting (**Table 2**).

A meta-analysis from 2017 studying the impact on the risk of cancer mortality and recurrence with RAAS blockers reported an improvement in disease-free survival for patients with urinary and colorectal cancer (in addition to pancreatic and prostate cancer) (73). A sub-analysis by specific drug class was not performed. No clinical trials involving patients with urinary tract or colorectal cancer and ARB intake have been completed; therefore, evidence is insufficient for the repurposing of ARBs as a treatment for these cancers. Concerning other cancers, two new clinical trials are recruiting for testing losartan among other drugs (**Table 2**).

Regarding adverse effects, the incidence of cough and angioedema is significantly lower than in patients treated with ARBs than in patients treated with ACEIs (61). However, the incidence of hypotension and hyperkalemia appears to be higher among individuals taking ARBs (101, 102). In a similar manner to ACEIs, ARBs are not recommended in the setting of nephrotoxicity, including drug-induced nephrotoxicity (78). The incidence of adverse effects of ARBs is unknown. In the trials with candesartan and losartan (in combination with gemcitabine and other chemotherapeutic drugs), the rate of hypotension varied from 6 to 40% (52, 58). In the gemcitabine-candesartan trial in patients with pancreatic cancer, hyperkalemia was reported in 6% of the patients, and reduced renal function (creatinine elevation) in 9% (58).

### Aldosterone Antagonists

Aldosterone antagonists, such as spironolactone and eplerenone, are recommended in combination with other antihypertensive drugs for the treatment of resistant hypertension. These drugs also belong to the pharmacological category of potassium-sparing diuretics.

#### Mechanism of Action

Physiologically, aldosterone exerts its effects upon binding to the mineralocorticoid receptor, an intracellular receptor identified in cells from several organs. Spironolactone and eplerenone competitively antagonize aldosterone binding to the mineralocorticoid receptor, and in the distal tubule of the nephron, this action leads to diuresis (103). Spironolactone has also been shown to bind to the androgen receptor, providing it with apparent antiandrogenic activity. At the same time, spironolactone has the ability to bind the progesterone receptor as an agonist (104). The spironolactone affinity for both androgen receptor and progesterone receptor is secondary to its structure, as this molecule is a derivative of progesterone (100). Eplerenone, on the contrary, was designed with this consideration in mind and has structural features that confer specificity for mineralocorticoid receptor (105). As a consequence of the no specificity of spironolactone, side effects related to its androgen receptor and progesterone receptor interaction include gynecomastia, breast pain and sexual dysfunction in men and menstrual irregularities in women (106–108). However, this antiandrogenic activity of spironolactone was considered to be of potential clinical utility for the treatment of prostate cancer.

#### Evidence From Studies *In Vitro* and in Animal Models

The antiandrogenic effect of spironolactone was first experimentally confirmed through radioactivity assays in prostatic tissue obtained from rats (104). Additionally, it was observed that spironolactone causes prostate weight reduction in this model (109, 110). However, in a posterior study, spironolactone exerted stimulatory activity in androgen-sensitive cells from a mouse mammary carcinoma model (111). This finding of partial agonist activity was later confirmed in a cell line specifically designed for testing androgen receptor transactivation (112). Furthermore, in addition to activating wild type androgen receptor, spironolactone has been observed to activate cells with point-variant androgen receptor that are commonly encountered in individuals with resistant prostate cancer (113). Finally, spironolactone was capable of negating the cytotoxic effects of the drug abiraterone, an inhibitor of CYP17 (and thus, synthesis of androgens) used in resistant forms of prostate cancer (114). Based on these reports, it appears that spironolactone acts as a partial AR agonist in androgen-depleted environments, such as that in patients treated for prostate cancer.

Other anticancer effects of spironolactone are described in **Table 1**. The hallmarks affected by spironolactone are avoiding immune destruction, activating invasion and metastasis and resisting cell death. Spironolactone also acts on an enabling characteristic of cancer that is genome instability through the inhibition of DNA damage repair (115). Spironolactone is capable of sensitizing cancer cells to platinum-base compounds (116).

#### Evidence From Clinical Studies

Initially, in the 1970s, spironolactone was reported to further reduce androgen levels in orchidectomized men with prostate cancer, suggesting that the drug could be useful as an adjuvant in these patients (117). This observation was reported in healthy men after the administration of the spironolactone derivative canrenone (118). More recently, a case report from France was published describing normalization of prostate-specific antigen in a patient with antecedent prostate cancer after treatment with spironolactone (119). In addition, epidemiological studies have correlated spironolactone intake with a reduced incidence of prostate cancer; all this evidence is in agreement with early observations of the antiandrogen activity of spironolactone (120, 121). In clinical practice, gynecomastia is a direct manifestation of this antiandrogen effect, and is, together with hyperkalemia, one of the most common adverse effects associated to spironolactone (122). However, several reports showing prostate cancer progression or treatment resistance after spironolactone initiation and resolving after spironolactone withdrawal have also been published (123–125). These accounts are better explained by the observation that spironolactone is a partial agonist rather than a pure antagonist of androgen receptor in androgen-depleted environments. Unfortunately, in addition to case reports, there are no observational or experimental studies analyzing the effects of spironolactone in individuals with prostatic cancer.

### β-Blockers

Expression of specific receptors able to bind ligands, transduce extracellular signals and activate intracellular signaling pathways is a key process in cells (119). β-blockers represent a heterogeneous pharmacological class with different pharmacodynamic properties and long-term effects on mortality and cardiovascular disease (126).

#### Mechanism of Action

The effect of these antihypertensive drugs occurs by blocking the action of endogenous catecholamines on the *β*-adrenergic receptor part of the autonomic nervous system, which is known to participate in blood pressure control (126). It has been suggested that β-blockers act on receptors associated with mechanisms that trigger tumorigenesis, angiogenesis and tumor metastasis (127). Some β-blockers such as propranolol, interfere with angiogenesis, including cell proliferation. β-AR antagonism also modulates the expression and activation of angiogenic signaling pathways, including angiopoietin/TIE2, VEGF, and hypoxia inducible factor. Additionally, propranolol exhibits a biphasic effect on vascular resistance, with low and high doses inducing vasoconstriction and vasodilation, respectively (127, 128).

There is evidence showing that the use of β*-*blockers, widespread to nonselective (carvedilol, labetalol, propranolol) and selective (β1-selective atenolol, nebivolol, metoprolol) agents, may have an important role in cancer treatment. However, the majority of preclinical studies have focused on the propranolol effect (129, 130).

#### Evidence From Studies *In Vitro* and Animal Models

It has been reported that propranolol activity reduces cell viability and migration in breast cancer cell lines, and the effect is increased when the drug is combined with metformin, another repurposed drug candidate. Combination of these drugs reduced tumor growth in two models of triple-negative breast cancer, improving survival. Additionally, the metastatic rate from breast cancer to distant metastasis was also attenuated, and the evidence suggests that propranolol abrogates the prometastatic process in tumor-bearing mice in dose-dependent antiproliferative and antiangiogenic effects *in vitro* (130, 131).

The traditional mechanism of action of β-blocker activity has been previously described, but in relation to cancer, the nonselective β-AR agonist isoproterenol increased activation of the ERK/MAPK pathway in pancreatic cancer cells (132). Propranolol and other β-blockers reduced the activity of MAPK in pancreatic cancer (29, 130, 133). In breast cancer, several alterations in tumor proliferation were observed in biopsies obtained from patients treated with propranolol, which may be related to propranolol administration. These findings were corroborated using the MDA-MB-231 breast cancer cell line, which was originally isolated from metastatic pleural effusion. Cell cycle analysis by flow cytometry in control and propranolol-treated breast cancer cells after 24 hours of treatment revealed important changes in cell viability (128). Taking into consideration previous evidence, propranolol may be considered a strategy given its inhibitory effects on the MAPK pathway to overcome resistance in melanoma treatment. Through inhibition of the MAPK pathway and other important pathways, β blockers act on several hallmarks of cancer (**Table 1**).

#### Evidence From Clinical Studies

In the assessment of the clinical usefulness of β-blockers in breast cancer, a meta-analysis from 2020 including 17 observational studies concluded that there was no association between these drugs and cancer recurrence, breast cancer-related deaths or all-cause deaths (134). However, in a phase II, placebo-controlled, randomized, triple-blind clinical trial, the authors observed that administration of propranolol prior to the resection of breast cancer was significantly associated with a decrease in expression of several metastasis markers (135). Thus, survival benefits derived from β-blockers should be evaluated in a phase III clinical trial.

In men with prostate cancer, a meta-analysis of 4 observational studies comprising 16,825 patients observed decreased prostate cancer-specific mortality associated with β-blockers (136). To date, no results from clinical trials assessing the effects of β-blockade in prostate cancer have been published.

Concerning pancreatic cancer, a population-based cohort study from Sweden observed that individuals with pancreatic ductal adenocarcinoma taking β-blockers exhibited a lower cancer-specific mortality after adjustment for other variables (137).

For ovarian cancer, an observational study by Watkins et al. including more than 1400 patients evaluating the impact of β-blockers found that these drugs increased median overall survival compared to individuals not taking drugs. Further stratification revealed that patients taking selective β-blockers fared no better than control individuals, and the increase in overall survival was specifically associated with nonselective β-blockers (138). A meta-analysis from 2018 by Yap et al., including two other studies (with a combined sample size smaller than the Watkins study), nonetheless found no net benefit for any β-blockers in ovarian cancer (140). Therefore, more studies are needed to clarify the impact of β-blockers in women suffering ovarian cancer.

In metastatic melanoma, nonselective β-blockers (in addition to specific therapy) were correlated with improved overall survival compared to selective β-blockers, as observed in a cohort study comprising 195 patients (139). In the meta-analysis by Yap et al., nonselective β-blockers were related to improved disease-free survival in melanoma as well; however, only two observational studies were included for melanoma (140). More recently, a small cohort study directly addressing propranolol reported a significant increase in progression-free survival among individuals taking this β-blocker (141). The addition of β-blockers was noted to improve progression-free survival in patients with lung adenocarcinoma with *EGFR* mutations receiving afatinib compared to patients taking afatinib without any β-blockers, as observed in a reanalysis of a phase III clinical trial (40). Immune checkpoint inhibitors could also benefit from the inclusion of β-blockers in the treatment of non-small cell lung carcinoma, as reported by an observational study including 109 patients (142).

For metastatic colorectal cancer, an observational study with 514 reported that β-blockers in combination with bevacizumab, a monoclonal antibody directed against VEGF-A, increased median overall survival and progression-free survival compared to treatment only with bevacizumab (143).

Perhaps one of the most interesting cases of the utility of β-blockers is angiosarcoma. Propranolol has been successfully used for at least a decade for the treatment of infantile hemangioma, a benign vascular neoplasm (144). This precedent and two case reports from 2015 describing excellent responses for propranolol in patients with angiosarcoma prompted two small trials studying its incorporation into chemotherapy for the treatment of advanced or metastatic angiosarcoma, which showed benefit in patients with this disease (41, 144–146). Even if the trials were small, totaling only 16 patients, cases of complete remission were observed (144).

β-blockers have shown benefits for patients with several types of cancers; however, with few exceptions, evidence comes from observational studies. Therefore, clinical trials are required to further establish the utility of this drug class in the management of individuals with cancer.

Adverse effects due to consumption of β-blockers are mainly related to beta blockade. Bradycardia, one of the most common side-effects is a direct consequence of the negative chronotropic activity of beta blockers. Other side-effects significatively associated with beta blockers are claudication, dizziness, diarrhea and hyperglycemia (147). Relative to RAAS blockers, beta blockers significantly increase the risk of fatigue, and they are associated with increased risk of sexual dysfunction in comparison to CCBs (148). The particular side-effects profile of beta blockers in patients with cancer is unknown.

### Calcium Channel Blockers (CCBs)

Calcium channels are attractive targets for the potential treatment of diseases, such as inflammatory or neuropathic pain, Parkinson’s disease and cancer (149). Voltage-gated Ca^2+^ channels are classified into at least five different subclasses (L-, N-, P, Q, and R type), and they have been targeted to treat hypertension, angina pectoris, and ventricular tachyarrhythmias (150).

#### Mechanism of Action

Calcium channel blockers inhibit the transmembrane flow of calcium by blocking L-type calcium ion channels, resulting in antagonism of vascular and myocardial smooth muscle, reduction of blood pressure, and coronary artery dilatation (150). Channel blocker drugs have been generally classified into two different groups according to their chemical structure: dihydropyridines, including amlodipine, bepridil, nifedipine, and nisoldipine; and nondihydropyridines, which include benzothiazepines (diltiazem) and phenylalkylamines (verapamil) (150).

The effect of calcium channel blockers in hypertension treatment is well known; however, it is not the only therapeutic effect. There is evidence reporting the antiproliferative action of this group of drugs in different neoplastic cell lines (151).

#### Evidence From *In Vitro* and Animal Model Studies

Since 1992, there have been several *in vivo* studies using L-type voltage-gated calcium channel blockers, such as amlodipine, diltiazem, and verapamil, all of which inhibit the proliferation of HT-39 human breast cancer cells with inhibitory concentration values ranging from 1.5 µM (for dihydropyridine amlodipine) to 10 µM (for phenylalkylamine verapamil) (145). Amlodipine inhibits proliferation in human epidermoid carcinoma by reducing BrDU incorporation into nucleic acids in serum-starved A431 cells (144). Verapamil has been associated with anticarcinogenic activity because it can inhibit P-glycoprotein, a protein associated with cancers with multidrug resistance phenotype when combined with chemotherapeutic agents due to its ability to promote intracellular drug accumulation (152).

Amlodipine is not the only CCB considered a possible alternative against cancer. Studies on verapamil showed that it has a direct effect on pancreatic cancer cells by inhibiting proliferation and inducing differentiation in human promyelocytic HL-60 cells. It has shown an inhibitory effect in human colonic tumor cells as well. Moreover, verapamil has shown antiproliferative effects in medulloblastoma, pineoblastoma, glioma, and neuroblastoma tumor cell lines (43, 153).

Diltiazem is another CCB normally used for treating hypertension; nevertheless, it is also considered an anticancer drug due to its effects on autophagy and apoptosis. In chemoresistant A549/D16 cells, diltiazem and verapamil have showed that both induce autophagy, and cotreatment with docetaxel or vincristine further enhances autophagy and apoptosis in typical and atypical chemoresistant lung cancer cells (16). The effects exerted by CCBs have been explained at the cellular level in several instances, and in a similar fashion to other antihypertensive drugs, they can be understood in the frame of the hallmarks of cancers, as shown in **Table 1**. Recently, amlodipine was reported to promote intracellular calcium entry through Orai1, a store-operated Ca^2+^ entry channel in glioblastoma cells. This resulted in the suppression of YAP/TAZ signaling, effectors of the Hippo pathway (32) which is related to several hallmarks of cancer (87).

Some characteristics and mechanisms related to treatment of cancer are to be understood as directly related to hallmarks of cancer. An important example is verapamil, which has been observed to re-sensitize chemoresistant cells. Multidrug resistance phenotype is commonly associated with increased expression of P-glycoprotein, a membrane transporter protein that is capable of extruding cytotoxic substances (154). Verapamil has been observed to reverse multidrug resistance phenotype in cancer cell lines (152), probably by acting directly at P-glycoprotein active sites (155). Verapamil is capable of reducing *MDR* (the gene encoding for P-glycoprotein) transcription as well (156). The evidence indicates that verapamil reverses chemoresistance in leukemia, colon cancer, hepatocellular carcinoma, and breast cancer cell lines (152, 157–159).

#### Evidence from Clinical Studies

Several studies have explored the impact of CCBs on survival in cancer patients. For instance, a small study by Takada et al. from 2019 observed that CCBs did not alter prognosis in patients with breast cancer; however, the time of exposure to CCBs was not taken into account (160). Another study from the United Kingdom that included more than 20,000 women with breast cancer reported no change in mortality after adjustment for other covariates (161).

The effects of CCBs in several other cancers have been studied. In patients with head and neck cancer, the use of CCBs was associated with a significantly higher risk of recurrence in a retrospective study (162). CCBs have also been associated with higher mortality in respiratory cancers in addition to higher all-cancer mortality in a Chinese study (163). CCB intake has been observed to be related to a worse outcome in individuals with acute myeloid leukemia (164). A phase I/II trial was completed evaluating the effect of verapamil in combination with hydroxyurea in patients with refractory meningioma. This trial included 7 patients, and no radiological response was observed after introduction of the treatment (165). Currently, a verapamil phase II trial in patients with Hodgkin lymphoma is recruiting (**Table 2**).

To date, pancreatic cancer is the disease with the most studies concerning the prognostic impact of CCBs. A retrospective study from the United Kingdom reported a survival benefit associated with CCBs and aspirin in combination in patients with pancreatic ductal adenocarcinoma after undergoing resection; neither CCBs nor aspirin alone were associated with improved overall survival in multivariate analysis (166). A subsequent study reported a longer overall survival in patients with unresectable pancreatic ductal adenocarcinoma taking CCBs alone in multivariate analysis (167). However, a previous work from a different group described a positive effect of CCBs on survival only in univariate analysis (168). These results suggest that CCBs could be repurposed for pancreatic cancer treatment; nonetheless, prospective studies are necessary to further understand the effect of CCBs in this disease.

After the discovery of the ability of verapamil to inhibit P-glycoprotein function *in vitro*, several trials in patients with chemoresistant cancers were started. Results on efficacy were not satisfactory, and reports of significant toxicity arose, thus, verapamil would not be tested in a phase III trial (169, 170).

As the other antihypertensive drug classes, CCBs are well tolerated by most patients. However, several adverse effects have been described after its consumption. For dihydropyridine CCBs, these side effects include headache, tachycardia, gastroesophageal reflux, peripheral edema and gingival hyperplasia. Non dihydropyridine CCBs common adverse effects are related to their higher activity in cardiac muscle, and those include bradycardia and atrioventricular block (171). Diltiazem and verapamil are known CYP3A4 inhibitors, henceforth, their administration is contraindicated in conjunction with drugs metabolized by this enzyme (e.g., sorafenib, sunitinib) (172).

## Diuretics

Diuretics work by increasing urinary output by decreasing net electrolyte and water reabsorption in different segments of the nephron, decreasing intravascular volume, and by decreasing vascular peripheral resistance (173). Although mechanisms regarding their role in oncology remain an area of active research, and limited studies are available, case-control studies and some reviews have associated some photosensitizing diuretics, such as thiazide and thiazide-like diuretics, besides their common adverse events such as orthostatic hypotension, hypokalemia, hyperglycemia and increase in uric acid concentration (173), with an increased risk for skin cancer (174). Other prospective studies have proposed that diuretics, such as furosemide plus spironolactone, may improve musculoskeletal symptoms induced by aromatase inhibitors in women with nonmetastatic breast cancer (175). Further studies are needed to provide more evidence to debate this matter.

## Discussion

In the present work, we summarized the preclinical and clinical evidence for the use of antihypertensive drugs belonging to 4 pharmacological categories in the management of several types of cancer. In several instances, potential anticancer activity is the result of the same mechanism of action that renders them useful for hypertension (shared biological pathways), such as in ARBs and ACEIs. For other drugs, such as propranolol or CCBs, this is the result of a different interaction not related to its hypotensive effect (drug pleiotropy). For practically all the drugs described here, their clinical usefulness in oncology is in combination with known chemotherapeutics, as they lack single-agent activity.

To date, the most frequently evaluated antihypertensive agents in the context of cancer belong to the β-blocker, ACEI and ARB pharmacological groups, whereas drugs from thiazide and thiazide-like diuretics remain less studied. This is likely a consequence of increasing understanding of the role of adrenergic receptors in cancer, as well as the role of the RAAS, especially the activity of the AT1R (18, 176). Nonetheless, the possibility of identifying new targets for pleiotropic drugs is present.

Drug repurposing has been regarded as a reasonable strategy for the development of new anticancer therapies, considering that the traditional development of oncology drugs has a phase I-to-FDA approval rate of 3.4%, one of the lowest compared to other therapeutic groups in recent years (177). Antihypertensive drugs are among the most prescribed drugs worldwide, most of them at an accessible price and with a well-known safety profile, making them of particular interest for drug repurposing. However, caution is advised. The majority of studies reporting a positive outcome related to the use of antihypertensive drugs in cancer patients are observational, which are more prone to bias that can lead to overestimation of their true effect. For instance, in a meta-analysis from 2016, Weberpals et al. found no evidence for an association between β-blockers and overall survival in patients with cancer after the exclusion of studies that may be affected by a specific bias known as immortal time bias. Other biases that need to be considered when assessing pharmacoepidemiologic studies (including studies of cancer outcomes and antihypertensive drugs) are confounding, selection and measurement biases (178). Therefore, randomized, blinded, placebo-controlled clinical trials are indispensable to evaluate a candidate drug for repurposing.

Only a few antihypertensive drugs have been evaluated with favorable results in clinical trials, as noted in this review, and just one of them in phase III, which was the combination of a β-blocker with afatinib in patients with lung adenocarcinoma and *EGFR* mutations. Currently, there are other 10 trials registered at clinicaltrials.gov in phase I/II on progress or completed but without published results, and 1 more trial with published results. These trials are mainly focused on pancreatic cancer, however, other tumors such as glioblastoma, breast cancer or osteosarcoma are also assessed in the remaining trials. One of the largest and most ambitious is the SMMART PRIME trial, in which information from multi-omics analysis of patients with different tumors will be used to test one or several among 55 drugs (including losartan). The results of these trials will be published in the coming years, thus informing what of those could progress to a phase III trial.

More rigorous phase III studies are necessary, the lack of financial incentives for pharmaceutical companies in the case of off-patent drugs imposes constraints on their design and funding. In light of these challenges, several approaches can be explored to refine drug candidates for repurposing. Computational approaches, such as pathway mapping, molecular docking and signature matching, can assist in the systematic (rather than serendipitous) prediction of new cancer targets for antihypertensive drugs (12). Better animal models (e.g., genetically engineered murine models) and patient-derived organoids can be used to more accurately predict the efficacy of a drug candidate at the preclinical stage (179, 180). Carefully designed observational studies considering selection bias and other biases are also necessary to more precisely identify the actual effects of these drugs. Finally, funding for all these efforts as well as phase II and phase III clinical trials could be primarily provided by public agencies and philanthropic organizations or *via* the creation of new financial incentives for industry, such as subsidies or tax credits (181).

In conclusion, a variety of antihypertensive drugs exhibit potential utility for repurposing as adjuvants in oncology, as observed in preclinical and clinical studies. However, higher quality evidence, particularly from randomized phase III clinical trials, is necessary to determine their impact in patients with cancer.

## Author Contributions

All authors listed have made direct and intellectual contribution to the present work. Original idea from TW-O and AR-C, both first authors made a substantial writing, and formatting contribution. All authors contributed to the article and approved the submitted version.

## Funding

JC-E (CVU 969754) and AR-C (CVU 963343) receive scholarship from CONACYT.

## Conflict of Interest

The authors declare that the research was conducted in the absence of any commercial or financial relationships that could be construed as a potential conflict of interest.

## References

[1] Bray F Ferlay J Soerjomataram I Siegel RL Torre LA Jemal A. Global Cancer Statistics 2018: GLOBOCAN Estimates of Incidence and Mortality Worldwide for 36 Cancers in 185 Countries. CA Cancer J Clin (2018) 68(6):394–424. 10.3322/caac.21492 30207593

[2] Sung H Ferlay J Siegel RL Laversanne M Soerjomataram I Jemal A. Global Cancer Statistics 2020: GLOBOCAN Estimates of Incidence and Mortality Worldwide for 36 Cancers in 185 Countries. CA Cancer J Clin (2021). 10.3322/caac.21660. in press. 33538338

[3] Fidler MM Gupta S Soerjomataram I Ferlay J Steliarova-Foucher E Bray F. Cancer Incidence and Mortality Among Young Adults Aged 20–39 Years Worldwide in 2012: A Population-Based Study. Lancet Oncol (2017) 18(12):1579–89. 10.1016/S1470-2045(17)30677-0 29111259

[4] National Cancer Institute. What Is Cancer? (2015). Available at: https://www.cancer.gov/about-cancer/understanding/what-is-cancer (Accessed 2020 Oct 19).

[5] Weinstein IB. The Origins of Human Cancer: Molecular Mechanisms of Carcinogenesis and Their Implications for Cancer Prevention and Treatment—Twenty-Seventh G. H. A. Clowes Memorial Award Lecture. Cancer Res (1988) 48(15):4135–43. 3292040

[6] Katalinić-Janković V Furci L Cirillo DM. Microbiology of Mycobacterium Tuberculosis and a New Diagnostic Test for TB. Eur Respir Monogr (2012) 58(June-2014):8–13. 10.1183/1025448x.10022311

[7] IARC Working Group on the Evaluation of Carcinogenic Risk to Humans. Some Chemicals Used as Solvents and in Polymer Manufacture. In: ARC Monographs on the Evaluation of Carcinogenic Risks to Humans Vol. 110. France: International Agency for Research on Cancer (2016). p. 1–276. 31829531

[8] Hanahan D Weinberg RA. Hallmarks of Cancer: The Next Generation. Cell (2011) 144(5):646–74. 10.1016/j.cell.2011.02.013 21376230

[9] Arruebo M Vilaboa N Sáez-Gutierrez B Lambea J Tres A Valladares M. Assessment of the Evolution of Cancer Treatment Therapies. Cancers (Basel) (2011) 3(3):3279–330. 10.3390/cancers3033279 PMC3759197 24212956

[10] Nowak-Sliwinska P Scapozza L Altaba A. Drug Repurposing in Oncology: Compounds, Pathways, Phenotypes and Computational Approaches for Colorectal Cancer. Biochim Biophys Acta - Rev Cancer (2019) 1871(2):434–54. 10.1016/j.bbcan.2019.04.005 PMC6528778 31034926

[11] Verbaanderd C Meheus L Huys I Pantziarka P. Repurposing Drugs in Oncology: Next Steps. Trends Cancer (2017) 3(8):543–6. 10.1016/j.trecan.2017.06.007 28780930

[12] Pushpakom S Iorio F Eyers PA Escott KJ Hopper S Wells A. Drug Repurposing: Progress, Challenges and Recommendations. Nat Rev Drug Discovery (2018) 18(1):41–58. 10.1038/nrd.2018.168 30310233

[13] Mills KT Stefanescu A He J. The Global Epidemiology of Hypertension. Nat Rev Nephrol (2020) 16(4):223–37. 10.1038/s41581-019-0244-2 PMC7998524 32024986

[14] Tsioufis C Thomopoulos C. Combination Drug Treatment in Hypertension. Pharmacol Res (2017) 125(Pt B):266–71. 10.1016/j.phrs.2017.09.011 28939201

[15] Yang Y Ma L Xu Y Liu Y Li W Cai J. Enalapril Overcomes Chemoresistance and Potentiates Antitumor Efficacy of 5-FU in Colorectal Cancer by Suppressing Proliferation, Angiogenesis, and NF-κb/STAT3-Regulated Proteins. Cell Death Dis (2020) 11(6). 10.1038/s41419-020-2675-x PMC7314775 32581212

[16] Wong BS Chiu LY Tu DG Sheu GT Chan TT. Anticancer Effects of Antihypertensive L-Type Calcium Channel Blockers on Chemoresistant Lung Cancer Cells Via Autophagy and Apoptosis. Cancer Manag Res (2020) 12:1913–27. 10.2147/CMAR.S228718 PMC7078713 32214849

[17] De Souza VB Silva EN Ribeiro ML De Martins WA. Hypertension in Patients With Cancer. Arq Bras Cardiol (2015) 104(3):246–52. 10.5935/abc.20150011 PMC4386854 25742420

[18] Wegman-Ostrosky T Soto-Reyes E Vidal-Millán S Sánchez-Corona J. The Renin-Angiotensin System Meets the Hallmarks of Cancer. JRAAS - J Renin-Angiotensin-Aldosterone Syst (2015) 16(2):227–33. 10.1177/1470320313496858 23934336

[19] George AJ Allen A Chand AL. Repurposing ARBs as Treatments for Breast Cancer. Aging (Albany NY) (2017) 9(5):1357–8. 10.18632/aging.101249 PMC5472736 28562314

[20] Blagosklonny MV. Carcinogenesis, Cancer Therapy and Chemoprevention. Cell Death Differ (2005) 12(6):592–602. 10.1038/sj.cdd.4401610 15818400

[21] Laurent S. Antihypertensive Drugs. Pharmacol Res (2017) 124:116–25. 10.1016/j.phrs.2017.07.026 28780421

[22] Rotshild V Azoulay L Zarifeh M Masarwa R Hirsh-Raccah B Perlman A. The Risk for Lung Cancer Incidence With Calcium Channel Blockers: A Systematic Review and Meta-Analysis of Observational Studies. Drug Saf (2018) 41(6):555–64. 10.1007/s40264-018-0644-4 29484611

[23] Wang Z White D Hoogeveen R Chen L Whitsel E Richardson P. Anti-Hypertensive Medication Use, Soluble Receptor for Glycation End Products and Risk of Pancreatic Cancer in the Women’s Health Initiative Study. J Clin Med (2018) 7(8):197. 10.3390/jcm7080197 PMC6111748 30072610

[24] Chen Q Zhang Q Zhong F Guo S Jin Z Shi W. Association Between Calcium Channel Blockers and Breast Cancer: A Meta-Analysis of Observational Studies. Pharmacoepidemiol Drug Saf (2014) 23:711–8. 10.1002/pds.3645 24829113

[25] Burnier M Narkiewicz K Kjeldsen SE Oparil S. New Data on Antihypertensive Drugs and Risk of Cancer: Should We Worry? Blood Press (2019) 28(1):1–3. 10.1080/08037051.2019.1568182 30714837

[26] Ni H Rui Q Zhu X Yu Z Gao R Liu H. Antihypertensive Drug Use and Breast Cancer Risk: A Metaanalysis of Observational Studies. Oncotarget (2017) 8(37):62545–60. 10.18632/oncotarget.19117 PMC5617528 28977968

[27] Grimaldi- Bensouda L Klungel O Kurz X de Groot MCH Maciel-Afonso AS de Bruin ML. Calcium Channel Blockers and Cancer: A Risk Analysis Using the UK Clinical Practice Research Datalink (Cprd). BMJ Open (2016) 6. 10.1136/bmjopen-2015-009147 PMC4716173 26747033

[28] Sanomachi T Suzuki S Togashi K Sugai A Seino S Okada M. Spironolactone, a Classic Potassium-Sparing Diuretic, Reduces Survivin Expression and Chemosensitizes Cancer Cells to Non-DNA-Damaging Anticancer Drugs. Cancer (2019) 11(10):1550. 10.3390/cancers11101550 PMC6826935 31614999

[29] Zhou C Chen X Zeng W Peng C Huang G Li X. Propranolol Induced G0/G1/S Phase Arrest and Apoptosis in Melanoma Cells Via AKT/MAPK Pathway. Oncotarget (2016) 7(42):68314–27. 10.18632/ONCOTARGET.11599 PMC5356557 27582542

[30] Zhang Y Cruickshanks N Yuan F Wang B Pahuski M Wulfkuhle J. Targetable T-type Calcium Channels Drive Glioblastoma. Cancer Res (2017) 77(13):3479–90. 10.1158/0008-5472.CAN-16-2347 PMC5505315 28512247

[31] Keir ST Friedman HS Reardon DA Bigner DD Gray LA. Mibefradil, a Novel Therapy for Glioblastoma Multiforme: Cell Cycle Synchronization and Interlaced Therapy in a Murine Model. J Neurooncol (2013) 111(2):97–102. 10.1007/s11060-012-0995-0 23086436

[32] Liu Z Wei Y Zhang L Yee PP Johnson M Zhang X. Induction of Store-Operated Calcium Entry (SOCE) Suppresses Glioblastoma Growth by Inhibiting the Hippo Pathway Transcriptional Coactivators YAP/TAZ. Oncogene (2019) 38(1):120–39. 10.1038/s41388-018-0425-7 PMC6318057 30082911

[33] Talarico G Orecchioni S Dallaglio K Reggiani F Mancuso P Calleri A. Aspirin and Atenolol Enhance Metformin Activity Against Breast Cancer by Targeting Both Neoplastic and Microenvironment Cells. Sci Rep (2016) 6(January):1–10. 10.1038/srep18673 26728433 PMC4700497

[34] Wei WJ Shen CT Song HJ Qiu ZL Luo QY. Propranolol Sensitizes Thyroid Cancer Cells to Cytotoxic Effect of Vemurafenib. Oncol Rep (2016) 36(3):1576–84. 10.3892/or.2016.4918 27432558

[35] De la Iglesia Iñigo S López-Jorge CE Gómez-Casares MT Lemes Castellano A Martín Cabrera P López Brito J. Induction of Apoptosis in Leukemic Cell Lines Treated With Captopril, Trandolapril and Losartan: A New Role in the Treatment of Leukaemia for These Agents. Leuk Res (2009) 33(6):810–6. 10.1016/j.leukres.2008.09.029 19010543

[36] Uemura H Ishiguro H Nakaigawa N Nagashima Y Miyoshi Y Fujinami K. Angiotensin II Receptor Blocker Shows Antiproliferative Activity in Prostate Cancer Cells: A Possibility of Tyrosine Kinase Inhibitor of Growth Factor. Mol Cancer Ther (2003) 2(11):1139–47. 14617787

[37] Saikawa S Kaji K Nishimura N Seki K Sato S Nakanishi K. Angiotensin Receptor Blockade Attenuates Cholangiocarcinoma Cell Growth by Inhibiting the Oncogenic Activity of Yes-associated Protein. Cancer Lett (2018) 434(April):120–9. 10.1016/j.canlet.2018.07.021 30031758

[38] Arrieta O Guevara P Escobar E García-Navarrete R Pineda B Sotelo J. Blockage of Angiotensin II Type I Receptor Decreases the Synthesis of Growth Factors and Induces Apoptosis in C6 Cultured Cells and C6 Rat Glioma. Br J Cancer (2005) 92(7):1247–52. 10.1038/sj.bjc.6602483 PMC2361987 15785746

[39] Zhang S Wang Y. Telmisartan Inhibits NSCLC A549 Cell Proliferation and Migration by Regulating the PI3K/AKT Signaling Pathway. Oncol Lett (2018) 15(4):5859–64. 10.3892/ol.2018.8002 PMC5840679 29552215

[40] Nilsson MB Sun H Diao L Tong P Liu D Li L. Stress Hormones Promote EGFR Inhibitor Resistance in NSCLC: Implications for Combinations With β-Blockers. Sci Transl Med (2017) 9(415). 10.1126/scitranslmed.aao4307 PMC5870120 29118262

[41] Amaya Cn Perkins M Belmont a Herrera C Nasrazadani a Vargas a. Non-selective Beta Blockers Inhibit Angiosarcoma Cell Viability and Increase Progression Free- and Overall-Survival in Patients Diagnosed With Metastatic Angiosarcoma. Oncoscience (2018) 5(3–4):109–19. 10.18632/oncoscience.413 PMC5978448 29854879

[42] Yoshida J Ishibashi T Yang M Nishio M. Amlodipine, a Ca2+ Channel Blocker, Suppresses Phosphorylation of Epidermal Growth Factor Receptor in Human Epidermoid Carcinoma A431 Cells. Life Sci (2010) 86(3–4):124–32. 10.1016/j.lfs.2009.11.014 19951711

[43] Zhang C Lv F Li Z Li X Wu XX Hoffman RM. Effect of Verapamil on the Expression of EGFR and NM23 in A549 Human Lung Cancer Cells. Anticancer Res (2009) 29(1):27–32. 19331130

[44] Saber S Mahmoud AAA Goda R Helal NS El-ahwany E Abdelghany RH. Perindopril, Fosinopril and Losartan Inhibited the Progression of Diethylnitrosamine-Induced Hepatocellular Carcinoma in Mice Via the Inactivation of Nuclear Transcription Factor Kappa-B. Toxicol Lett (2018) 295(January):32–40. 10.1016/j.toxlet.2018.05.036 29859236

[45] Yoshida J Ishibashi T Nishio M. G1 Cell Cycle Arrest by Amlodipine, a Dihydropyridine Ca2+ Channel Blocker, in Human Epidermoid Carcinoma A431 Cells. Biochem Pharmacol (2007) 73(7):943–53. 10.1016/j.bcp.2006.12.011 17217918

[46] Shrestha S Noh JM Kim SY Ham HY Kim YJ Yun YJ. Angiotensin Converting Enzyme Inhibitors and Angiotensin II Receptor Antagonist Attenuate Tumor Growth Via Polarization of Neutrophils Toward an Antitumor Phenotype. Oncoimmunology (2016) 5(1):1–14. 10.1080/2162402X.2015.1067744 PMC4760329 26942086

[47] Vallejo Ardila DL Walsh KA Fifis T Paolini R Kastrappis G Christophi C. Immunomodulatory Effects of Renin-Angiotensin System Inhibitors on T Lymphocytes in Mice With Colorectal Liver Metastases. J Immunother Cancer (2020) 8(1):1–10. 10.1136/jitc-2019-000487 PMC7253054 32448803

[48] Xie G Cheng T Lin J Zhang L Zheng J Liu Y. Local Angiotensin II Contributes to Tumor Resistance to Checkpoint Immunotherapy. J Immunother Cancer (2018) 6(1):1–14. 10.1186/s40425-018-0401-3 30208943 PMC6134794

[49] Leung WH Vong QP Lin W Janke L Chen T Leung W. Modulation of NKG2D Ligand Expression and Metastasis in Tumors by Spironolactone Via Rxrγ Activation. J Exp Med (2013) 210(12):2675–92. 10.1084/jem.20122292 PMC3832934 24190430

[50] Liao P Song K Zhu Z Liu Z Zhang W Li W. Propranolol Suppresses the Growth of Colorectal Cancer Through Simultaneously Activating Autologous Cd8+ T Cells and Inhibiting Tumor AKT/MAPK Pathway. Clin Pharmacol Ther (2020) 108(3):606–15. 10.1002/cpt.1894 32418204

[51] Prontera C Mariani B Rossi C Poggi A Rotilio D. Inhibition of Gelatinase A (Mmp-2) by Batimastat and Captopril Reduces Tumor Growth and Lung Metastases in Mice Bearing Lewis Lung Carcinoma. Int J Cancer (1999) 81(5):761–6. 10.1002/(SICI)1097-0215(19990531)81:5<761::AID-IJC16>3.0.CO;2-1 10328230

[52] Murphy JE Wo JY Ryan DP Clark JW Jiang W Yeap BY. Total Neoadjuvant Therapy With FOLFIRINOX in Combination With Losartan Followed by Chemoradiotherapy for Locally Advanced Pancreatic Cancer: A Phase 2 Clinical Trial. JAMA Oncol (2019) 5(7):1020–7. 10.1001/jamaoncol.2019.0892 PMC6547247 31145418

[53] Sloan EK Priceman SJ Cox BF Yu S Pimentel MA Tangkanangnukul V. The Sympathetic Nervous System Induces a Metastatic Switch in Primary Breast Cancer. Cancer Res (2010) 70(18):7042–52. 10.1158/0008-5472.CAN-10-0522 PMC2940980 20823155

[54] Zhang D Ma QY Hu HT Zhang M. β2-Adrenergic Antagonists Suppress Pancreatic Cancer Cell Invasion by Inhibiting CREB, Nfκb and AP-1. Cancer Biol Ther (2010) 10(1):19–29. 10.4161/cbt.10.1.11944 20424515

[55] Jacquemet G Baghirov H Georgiadou M Sihto H Peuhu E Cettour-Janet P. L-type calcium channels regulate filopodia stability and cancer cell Invasion Downstream of Integrin Signalling. Nat Commun (2016) 7(7491). 10.1038/ncomms13297 PMC5146291 27910855

[56] Pinter M Jain RK. Targeting the Renin-Angiotensin System to Improve Cancer Treatment: Implications for Immunotherapy. Sci Transl Med (2017) 9(410). 10.1126/scitranslmed.aan5616 PMC5928511 28978752

[57] Tatokoro M Fujii Y Kawakami S Saito K Koga F Matsuoka Y. Phase-II Trial of Combination Treatment of Interferon-α, Cimetidine, Cyclooxygenase-2 Inhibitor and Renin-Angiotensin-System Inhibitor (I-CCA Therapy) for Advanced Renal Cell Carcinoma. Cancer Sci (2011) 102(1):137–43. 10.1111/j.1349-7006.2010.01756.x 20973869

[58] Nakai Y Isayama H Ijichi H Sasaki T Takahara N Ito Y. A Multicenter Phase II Trial of Gemcitabine and Candesartan Combination Therapy in Patients With Advanced Pancreatic Cancer: GECA2. Invest New Drugs (2013) 31(5):1294–9. 10.1007/s10637-013-9972-5 23690239

[59] Abd-Alhaseeb MM Zaitone SA Abou-El-Ela SH Moustafa YM. Olmesartan Potentiates the Anti-Angiogenic Effect of Sorafenib in Mice Bearing Ehrlich’s Ascites Carcinoma: Role of Angiotensin (1-7). PloS One (2014) 9(1):1–10. 10.1371/journal.pone.0085891 PMC3899087 24465768

[60] Solomon CG Taler SJ. Initial Treatment of Hypertension. N Engl J Med (2018) 378(7):636–44. 10.1056/NEJMcp1613481 29443671

[61] Messerli FH Bangalore S Bavishi C Rimoldi SF. Angiotensin-Converting Enzyme Inhibitors in Hypertension: to Use or Not to Use? J Am Coll Cardiol (2018) 71(13):1474–82. 10.1016/j.jacc.2018.01.058 29598869

[62] Tirupula KC Desnoyer R Speth RC Karnik SS. Atypical Signaling and Functional Desensitization Response of MAS Receptor to Peptide Ligands. PloS One (2014) 9(7). 10.1371/journal.pone.0103520 PMC4113456 25068582

[63] Dhanachandra Singh K Karnik SS. Angiotensin Receptors: Structure, Function, Signaling and Clinical Applications. J Cell Signal (2017) 01(02):1–8. 10.4172/2576-1471.1000111 PMC4976824 27512731

[64] Ferrario CM Trask AJ Jessup JA. Advances in Biochemical and Functional Roles of Angiotensin-Converting Enzyme 2 and Angiotensin-(1-7) in Regulation of Cardiovascular Function. Am J Physiol - Hear Circ Physiol (2005) 289(6):658–66. 10.1152/ajpheart.00618.2005 PMC7203566 16055515

[65] Xia H Lazartigues E. Angiotensin-Converting Enzyme 2: Central Regulator for Cardiovascular Function. Curr Hypertens Rep (2010) 12(3):170–5. 10.1007/s11906-010-0105-7 PMC3093757 20424953

[66] Tipnis SR Hooper NM Hyde R Karran E Christie G Turner AJ. A Human Homolog of Angiotensin-Converting Enzyme: Cloning and Functional Expression as a Captopril-Insensitive Carboxypeptidase. J Biol Chem (2000) 275(43):33238–43. 10.1074/jbc.M002615200 10924499

[67] Kubota M Shimizu M Sakai H Yasuda Y Ohno T Kochi T. Renin-Angiotensin System Inhibitors Suppress Azoxymethane-Induced Colonic Preneoplastic Lesions in C57BL/KsJ-db/db Obese Mice. Biochem Biophys Res Commun (2011) 410(1):108–13. 10.1016/j.bbrc.2011.05.115 21640075

[68] Fendrich V Lopez CL Manoharan J Maschuw K Wichmann S Baier A. Enalapril and ASS Inhibit Tumor Growth in a Transgenic Mouse Model of Islet Cell Tumors. Endocr Relat Cancer (2014) 21(5):813–24. 10.1530/ERC-14-0175 25121552

[69] Fendrich V Chen NM Neef M Waldmann J Buchholz M Feldmann G. The angiotensin-I-converting Enzyme Inhibitor Enalapril and Aspirin Delay Progression of Pancreatic Intraepithelial Neoplasia and Cancer Formation in a Genetically Engineered Mouse Model of Pancreatic Cancer. Gut (2010) 59(5):630–7. 10.1136/gut.2009.188961 19880966

[70] Araújo WF Naves MA Ravanini JN Schor N Teixeira VPC. Renin-Angiotensin System (RAS) Blockade Attenuates Growth and Metastatic Potential of Renal Cell Carcinoma in Mice. Urol Oncol Semin Orig Investig (2015) 33(9):389.e1–7. 10.1016/j.urolonc.2014.11.022 25595575

[71] Liu C Zhang JW Hu L Song YC Zhou L Fan Y. Activation of the AT1R/HIF-1 α /Ace Axis Mediates Angiotensin Ii-Induced VEGF Synthesis in Mesenchymal Stem Cells. BioMed Res Int (2014) 2014. 10.1155/2014/627380 PMC4221905 25401104

[72] Mc Menamin ÚC Murray LJ Cantwell MM Hughes CM. Angiotensin-Converting Enzyme Inhibitors and Angiotensin Receptor Blockers in Cancer Progression and Survival: A Systematic Review. Cancer Causes Control (2012) 23(2):221–30. 10.1007/s10552-011-9881-x 22116540

[73] Sun H Li T Zhuang R Cai W Zheng Y. Do Renin-Angiotensin System Inhibitors Influence the Recurrence, Metastasis, and Survival in Cancer Patients? Evidence From a Meta-Analysis Including 55 Studies. Med (United States) (2017) 96(13). 10.1097/MD.0000000000006394 PMC5380250 28353566

[74] Liu H Naxerova K Pinter M Incio J Lee H Shigeta K. Use of Angiotensin System Inhibitors is Associated With Immune Activation and Longer Survival in Nonmetastatic Pancreatic Ductal Adenocarcinoma. Clin Cancer Res (2017) 23(19):5959–69. 10.1158/1078-0432.CCR-17-0256 PMC5856249 28600474

[75] Wilop S Von Hobe S Crysandt M Esser A Osieka R Jost E. Impact of Angiotensin I Converting Enzyme Inhibitors and Angiotensin II Type 1 Receptor Blockers on Survival in Patients With Advanced non-Small-Cell Lung Cancer Undergoing First-Line Platinum-Based Chemotherapy. J Cancer Res Clin Oncol (2009) 135(10):1429–35. 10.1007/s00432-009-0587-3 PMC12160257 19399518

[76] Barone M Viggiani MT Losurdo G Principi M Di LA. Systematic Review: Renin-angiotensin System Inhibitors in Chemoprevention of Hepatocellular Carcinoma. World J Gastroenterol (2019) 25(20):2524–38. 10.3748/wjg.v25.i20.2524 PMC6543242 31171895

[77] Blaes A Duprez D Defor T Shanley R Beckwith H Haddad T. Angiotensin Converting Enzyme Inhibitors (ACEI) and Doxorubicin Pharmacokinetics in Women Receiving Adjuvant Breast Cancer Treatment. Springerplus (2015) 4:32. 10.1186/s40064-015-0802-4 25646154 PMC4309801

[78] Komaki K Kusaba T Tanaka M Kado H Shiotsu Y Matsui M. Lower Blood Pressure and Risk of Cisplatin Nephrotoxicity: A Retrospective Cohort Study. BMC Cancer (2017) 17(1):1–8. 10.1186/s12885-017-3135-6 28219368 PMC5319111

[79] Bonanni L Dalla Vestra M. Oral Renin Inhibitors in Clinical Practice: A Perspective Review. Ther Adv Chronic Dis (2012) 3(4):173–81. 10.1177/2040622312446244 PMC3539287 23342233

[80] Wang C Guo D Wang Q You S Qiao Z Liu Y. Aliskiren Targets Multiple Systems to Alleviate Cancer Cachexia. Oncol Rep (2016) 36(5):3014–22. 10.3892/or.2016.5118 27667116

[81] Dézsi CA. The Different Therapeutic Choices With ARBs. Which One to Give? When? Why? Am J Cardiovasc Drugs (2016) 16(4):255–66. 10.1007/s40256-016-0165-4 PMC4947116 26940560

[82] Shim J Song W Morice AH. Drug-Induced Cough. Physiol Res (2020) 69:S81–92. 10.33549/physiolres.934406 PMC8604055 32228014

[83] Woo Y Jung YJ. Angiotensin II Receptor Blockers Induce Autophagy in Prostate Cancer Cells. Oncol Lett (2017) 13(5):3579–85. 10.3892/ol.2017.5872 PMC5431597 28529582

[84] Samukawa E Fujihara S Oura K Iwama H Yamana Y Tadokoro T. Angiotensin Receptor Blocker Telmisartan Inhibits Cell Proliferation and Tumor Growth of Cholangiocarcinoma Through Cell Cycle Arrest. Int J Oncol (2017) 51(6):1674–84. 10.3892/ijo.2017.4177 PMC5673010 29075786

[85] Matsui T Chiyo T Kobara H Fujihara S Fujita K Namima D. Telmisartan Inhibits Cell Proliferation and Tumor Growth of Esophageal Squamous Cell Carcinoma by Inducing S-phase Arrest In Vitro and In Vivo. Int J Mol Sci (2019) 20(13). 10.3390/ijms20133197 PMC6651359 31261874

[86] Leitão Oliveira ALC de Melo Silveira RF de Oliveira Rocha HA de França Cavalcanti P de Araújo AA. Telmisartan Induces Apoptosis and Regulates Bcl-2 in Human Renal Cancer Cells. Exp Biol Med (2015) 240(1):34–44. 10.1177/1535370214546267 PMC4935194 25125501

[87] Calses PC Crawford JJ Lill JR Dey A. Hippo Pathway in Cancer: Aberrant Regulation and Therapeutic Opportunities. Trends Cancer (2019) 5(5):297–307. 10.1016/j.trecan.2019.04.001 31174842

[88] Pfeffer MA Swedberg K Granger CB Held P McMurray JJV Michelson EL. Effects of Candesartan on Mortality and Morbidity in Patients With Chronic Heart Failure: The CHARM-Overall Programme. Lancet (2003) 362(9386):759–66. 10.1016/S0140-6736(03)14282-1 13678868

[89] Teo KK. Effects of Telmisartan, Irbesartan, Valsartan, Candesartan, and Losartan on Cancers in 15 Trials Enrolling 138 769 Individuals. J Hypertens (2011) 29(4):623–35. 10.1097/HJH.0b013e328344a7de 21358417

[90] Cho MA Jeong SY Sohn I Kim MS Kang JH Paik ES. Impact of Angiotensin Receptor Blockers, Beta Blockers, Calcium Channel Blockers and Thiazide Diuretics on Survival of Ovarian Cancer Patients. Cancer Res Treat (2020) 52(2):645–54. 10.4143/crt.2019.509 PMC7176948 32019281

[91] Zhao Y Cao J Melamed A Worley M Gockley A Jones D. Losartan Treatment Enhances Chemotherapy Efficacy and Reduces Ascites in Ovarian Cancer Models by Normalizing the Tumor Stroma. Proc Natl Acad Sci USA (2019) 116(6):2210–9. 10.1073/pnas.1818357116 PMC6369817 30659155

[92] Uemura H Hasumi H Kawahara T Sugiura S Miyoshi Y Nakaigawa N. Pilot Study of Angiotensin II Receptor Blocker in Advanced Hormone-Refractory Prostate Cancer. Int J Clin Oncol (2005) 10(6):405–10. 10.1007/s10147-005-0520-y 16369744

[93] Santala EEE Rannikko A Murtola TJ. Antihypertensive Drugs and Prostate Cancer Survival After Radical Prostatectomy in Finland—A Nationwide Cohort Study. Int J Cancer (2019) 144(3):440–7. 10.1002/ijc.31802 30110124

[94] Siltari A Siltari A Murtola TJ Murtola TJ Talala K Taari K. Antihypertensive Drug Use and Prostate Cancer-Specific Mortality in Finnish Men. PloS One (2020) 15(6). 10.1371/journal.pone.0234269 PMC7323967 32598349

[95] Nakai Y Isayama H Ijichi H Sasaki T Sasahira N Hirano K. Inhibition of Renin-Angiotensin System Affects Prognosis of Advanced Pancreatic Cancer Receiving Gemcitabine. Br J Cancer (2010) 103(11):1644–8. 10.1038/sj.bjc.6605955 PMC2994224 20978506

[96] Nakai Y Isayama H Ijichi H Sasaki T Kogure H Yagioka H. Phase I Trial of Gemcitabine and Candesartan Combination Therapy in Normotensive Patients With Advanced Pancreatic Cancer: GECA1. Cancer Sci (2012) 103(8):1489–92. 10.1111/j.1349-7006.2012.02311.x PMC7659287 22515232

[97] Murphy JE Wo JY Ryan DP Jiang W Yeap BY Drapek LC. Total Neoadjuvant Therapy With FOLFIRINOX Followed by Individualized Chemoradiotherapy for Borderline Resectable Pancreatic Adenocarcinoma: A Phase 2 Clinical Trial. JAMA Oncol (2018) 4(7):963–9. 10.1001/jamaoncol.2018.0329 PMC6145728 29800971

[98] Carpentier AF Ferrari D Bailon O Ursu R Banissi C Dubessy AL. Steroid-Sparing Effects of angiotensin-II Inhibitors in Glioblastoma Patients. Eur J Neurol (2012) 19(10):1337–42. 10.1111/j.1468-1331.2012.03766.x 22650322

[99] Kourilsky A Bertrand G Ursu R Doridam J Barlog C Faillot T. Impact of Angiotensin-II Receptor Blockers on Vasogenic Edema in Glioblastoma Patients. J Neurol (2016) 263(3):524–30. 10.1007/s00415-015-8016-9 26754004

[100] Ursu R Thomas L Psimaras D Chinot O Le Rhun E Ricard D. Angiotensin II Receptor Blockers, Steroids and Radiotherapy in Glioblastoma—a Randomised Multicentre Trial (ASTER Trial). An ANOCEF Study. Eur J Cancer (2019) 109:129–36. 10.1016/j.ejca.2018.12.025 30716716

[101] Yusuf S Teo KK Pogue J Dyal L Copland I Schumacher H. Telmisartan, Ramipril, or Both in Patients At High Risk for Vascular Events. N Engl J Med (2008) 358(15):1547–59. 10.1056/NEJMoa0801317 18378520

[102] Sadjadi SA McMillan JI Jaipaul N Blakely P Hline SS. A Comparative Study of the Prevalence of Hyperkalemia With the Use of Angiotensin Converting Enzyme Inhibitors Versus Angiotensin Receptor Blockers. Ther Clin Risk Manag (2009) 5(1):547–52. 10.2147/tcrm.s5176 PMC2710386 19707264

[103] Guichard JL Clark D Calhoun DA Ahmed MI. Aldosterone Receptor Antagonists: Current Perspectives and Therapies. Vasc Health Risk Manag (2013) 9(1):321–31. 10.2147/VHRM.S33759 PMC3699348 23836977

[104] Corvol P Michaud A Menard J Freifeld M Mahoudeau J. Antiandrogenic Effect of Spirolactones: Mechanism of Action. Endocrinology (1975) 97(1):52–8. 10.1210/endo-97-1-52 166833

[105] Garthwaite SM McMahon EG. The Evolution of Aldosterone Antagonists. Mol Cell Endocrinol (2004) 217(1–2):27–31. 10.1016/j.mce.2003.10.005 15134797

[106] Pitt B Zannad F Remme WJ Cody R Castaigne A Perez A. The Effect of Spironolactone on Morbidity and Mortality in Patients With Severe Heart Failure. N Engl J Med (1999) 341(10):709–17. 10.1056/nejm199909023411001 10471456

[107] Engbaek M Hjerrild M Hallas J Jacobsen IA. The Effect of Low-Dose Spironolactone on Resistant Hypertension. J Am Soc Hypertens (2010) 4(6):290–4. 10.1016/j.jash.2010.10.001 21130975

[108] Armanini D Andrisani A Bordin L Sabbadin C. Spironolactone in the Treatment of Polycystic Ovary Syndrome. Expert Opin Pharmacother (2016) 17(13):1713–5. 10.1080/14656566.2016.1215430 27450358

[109] Basinger GT Gittes RF. Antiandrogenic Effect of Spironolactone in Rats. J Urol (1974) 111(1):77–80. 10.1016/S0022-5347(17)59893-6 4813557

[110] Carter DB Silverberg AB Harris SE. Effect of Spironolactone on Androgen-Dependent Proteins in the Ventral Prostate of the Rat. J Endocrinol (1980) 86(3):471–6. 10.1677/joe.0.0860471 7430906

[111] Labrie F Veilleux R. A Wide Range of Sensitivities to Androgens Develops in Cloned Shionogi Mouse Mammary Tumor Cells. Prostate (1986) 8(3):293–300. 10.1002/pros.2990080309 3703748

[112] Térouanne B Tahiri B Georget V Belon C Poujol N Avances C. A Stable Prostatic Bioluminescent Cell Line to Investigate Androgen and Antiandrogen Effects. Mol Cell Endocrinol (2000) 160(1–2):39–49. 10.1016/S0303-7207(99)00251-8 10715537

[113] Richards J Lim AC Hay CW Taylor AE Wingate A Nowakowska K. Interactions of Abiraterone, Eplerenone, and Prednisolone With Wild-Type and Mutant Androgen Receptor: A Rationale for Increasing Abiraterone Exposure or Combining With MDV3100. Cancer Res (2012) 72(9):2176–82. 10.1158/0008-5472.CAN-11-3980 PMC4281708 22411952

[114] Bedussi F Galli D Fragni M Valcamonico F Rossini E Dalla Volta A. Amiloride Is Effective in the Management of Abiraterone-Induced Mineralocorticoid Excess Syndrome Without Interfering With Its Antineoplastic Activity. Pharmacology (2017) 100(5–6):261–8. 10.1159/000477547 28797006

[115] Shahar OD Kalousi A Eini L Fisher B Weiss A Darr J. A High-Throughput Chemical Screen With FDA Approved Drugs Reveals That the Antihypertensive Drug Spironolactone Impairs Cancer Cell Survival by Inhibiting Homology Directed Repair. Nucleic Acids Res (2014) 42(9):5689–701. 10.1093/nar/gku217 PMC4027216 24682826

[116] Zhang Z Zhou L Xie N Nice EC Zhang T Cui Y. Overcoming Cancer Therapeutic Bottleneck by Drug Repurposing. Signal Transduct Target Ther (2020) 5(1). 10.1038/s41392-020-00213-8 PMC7331117 32616710

[117] Walsh PC Siiteri PK. Suppression of Plasma Androgens by Spironolactone in Castrated Men With Carcinoma of the Prostate. J Urol (1975) 114(2):254–6. 10.1016/S0022-5347(17)67001-0 125803

[118] Tidd MJ Horth CE Ramsay LE Shelton JR Palmer RF. Endocrine Effects of Spironolactone in Man. Clin Endocrinol (Oxf) (1978) 9(5):389–99. 10.1111/j.1365-2265.1978.tb03578.x 152681

[119] Rybikowski S Maurin C Deturmeny J Delaporte V Lechevallier E Coulange C. PSA Et Spironolactone. Prog en Urol (2010) 20(2):154–7. 10.1016/j.purol.2009.04.002 20142058

[120] Beckmann K Garmo H Lindahl B Holmberg L Stattin P Adolfsson J. Spironolactone Use is Associated With Lower Prostate Cancer Risk: A Population-Wide Case-Control Study. Prostate Cancer Prostatic Dis (2020) 23(3):527–33. 10.1038/s41391-020-0220-8 32123316

[121] Mackenzie IS Morant SV Wei L Thompson AM MacDonald TM. Spironolactone Use and Risk of Incident Cancers: A Retrospective, Matched Cohort Study. Br J Clin Pharmacol (2017) 83(3):653–63. 10.1111/bcp.13152 PMC5306481 27735065

[122] Williams EM Katholi RE Karambelas MR. Use and Side-Effect Profile of Spironolactone in a Private Cardiologist’s Practice. Clin Cardiol (2006) 29(4):149–53. 10.1002/clc.4960290405 PMC6654241 16649723

[123] Dhondt B Buelens S Van Besien J Beysens M De Bleser E Ost P. Abiraterone and Spironolactone in Prostate Cancer: A Combination to Avoid. Acta Clin Belgica Int J Clin Lab Med (2019) 74(6):439–44. 10.1080/17843286.2018.1543827 30477405

[124] Sundar S Dickinson PD. Spironolactone, a Possible Selective Androgen Receptor Modulator, Should be Used With Caution in Patients With Metastatic Carcinoma of the Prostate. BMJ Case Rep (2012) 2012. 10.1136/bcr.11.2011.5238 PMC3291010 22665559

[125] Flynn T Guancial EA Kilari M Kilari D. Case Report: Spironolactone Withdrawal Associated With a Dramatic Response in a Patient With Metastatic Castrate-Resistant Prostate Cancer. Clin Genitourin Cancer (2017) 15(1):e95–7. 10.1016/j.clgc.2016.08.006 27641657

[126] Wiysonge CS Bradley HA JV Mayosi BM Mbewu A Opie LH. Beta-Blockers for Hypertension ( Review ) Beta-blockers for Hypertension. Wiysonge CS, Editor. Cochrane Database Syst Rev (2012) 11(8):CD002003. 10.1002/14651858.CD002003.pub3 23152211

[127] Peixoto R Pereira M de L Oliveira M. Beta-Blockers and Cancer: Where are We? Pharmaceuticals (2020) 13(6). 10.3390/ph13060105 PMC7345088 32466499

[128] Montoya A Varela-Ramirez A Dickerson E Pasquier E Torabi A Aguilera R. The Beta Adrenergic Receptor Antagonist Propranolol Alters Mitogenic and Apoptotic Signaling in Late Stage Breast Cancer. BioMed J (2019) 42(3):155–65. 10.1016/j.bj.2019.02.003 PMC6717753 31466709

[129] Dezong G Zhongbing M Qinye F Zhigang Y. Carvedilol Suppresses Migration and Invasion of Malignant Breast Cells by Inactivating Src Involving cAMP/PKA and Pkcδ Signaling Pathway. J Cancer Res Ther (2014) 10(4):991–7. 10.4103/0973-1482.137664 25579542

[130] Pantziarka P Bryan BA Crispino S Dickerson EB. Propranolol and Breast Cancer—a Work in Progress. Ecancermedicalscience (2018) 12. 10.3332/ecancer.2018.ed82 PMC6027968 30034523

[131] Pasquier E Ciccolini J Carre M Giacometti S Fanciullino R Pouchy C. Propranolol Potentiates the Anti-Angiogenic Effects and Antitumor Efficacy of Chemotherapy Agents: Implication in Breast Cancer Treatment. Oncotarget (2011) 2(10):797–809. 10.18632/oncotarget.343 22006582 PMC3248157

[132] Lin X Luo K Lv Z Huang J. Beta-Adrenoceptor Action on Pancreatic Cancer Cell Proliferation and Tumor Growth in Mice. Hepatogastroenterology (2012) 59(114):584–8. 10.5754/hge11271 22353526

[133] Zhang D Ma Q Wang Z Zhang M Guo K Wang F. β 2-Adrenoceptor Blockage Induces G 1/s Phase Arrest and Apoptosis in Pancreatic Cancer Cells Via Ras/Akt/Nfκb Pathway. Mol Cancer (2011) 10. 10.1186/1476-4598-10-146 PMC3250953 22118662

[134] Li C Li T Tang R Yuan S Zhang W. β-Blocker Use is Not Associated With Improved Clinical Outcomes in Women With Breast Cancer: A Meta-Analysis. Biosci Rep (2020) 40(6). 10.1042/BSR20200721 PMC7303345 32436935

[135] Hiller JG Cole SW Crone EM Byrne DJ Shackleford DM Pang JMB. Preoperative β-Blockade With Propranolol Reduces Biomarkers of Metastasis in Breast Cancer: A Phase II Randomized Trial. Clin Cancer Res (2020) 26(8):1803–11. 10.1158/1078-0432.CCR-19-2641 31754048

[136] Lu H Liu X Guo F Tan S Wang G Liu H. Impact of Beta-Blockers on Prostate Cancer Mortality: A Meta-Analysis of 16,825 Patients. Onco Targets Ther (2015) 8:985–90. 10.2147/OTT.S78836 PMC4425323 25995645

[137] Udumyan R Montgomery S Fang F Almroth H Valdimarsdottir U Ekbom A. Beta-Blocker Drug Use and Survival Among Patients With Pancreatic Adenocarcinoma. Cancer Res (2017) 77(13):3700–7. 10.1158/0008-5472.CAN-17-0108 28473530

[138] Watkins JL Thaker PH Nick AM Ramondetta LM Kumar S Urbauer DL. Clinical Impact of Selective and Nonselective Beta-Blockers on Survival in Patients With Ovarian Cancer. Cancer (2015) 121(19):3444–51. 10.1002/cncr.29392 PMC4575637 26301456

[139] Kokolus KM Zhang Y Sivik JM Schmeck C Zhu J Repasky EA. Beta Blocker Use Correlates With Better Overall Survival in Metastatic Melanoma Patients and Improves the Efficacy of Immunotherapies in Mice. Oncoimmunology (2018) 7(3). 10.1080/2162402X.2017.1405205 PMC5790362 29399407

[140] Yap A Lopez-Olivo MA Dubowitz J Pratt G Hiller J Gottumukkala V. Effect of Beta-Blockers on Cancer Recurrence and Survival: A Meta-Analysis of Epidemiological and Perioperative Studies. Br J Anaesth (2018) 121(1):45–57. 10.1016/j.bja.2018.03.024 29935594

[141] De Giorgi V Grazzini M Benemei S Marchionni N Botteri E Pennacchioli E. Propranolol for Off-Label Treatment of Patients With Melanoma: Results From a Cohort Study. JAMA Oncol (2018) 4(2). 10.1001/jamaoncol.2017.2908 PMC5838568 28973254

[142] Oh MS Guzner A Wainwright DA Mohindra NA Chae YK Behdad A. The Impact of Beta Blockers on Survival Outcomes in Patients With Non–Small-Cell Lung Cancer Treated With Immune Checkpoint Inhibitors. Clin Lung Cancer (2021) 22(1):e57–62. 10.1016/j.cllc.2020.07.016 PMC7785632 32900613

[143] Fiala O Ostasov P Sorejs O Liska V Buchler T Poprach A. Incidental Use of Beta-Blockers is Associated With Outcome of Metastatic Colorectal Cancer Patients Treated With Bevacizumab-Based Therapy: A Single-Institution Retrospective Analysis of 514 Patients. Cancers (Basel) (2019) 11(12). 10.3390/Cancers11121856 PMC6966537 31769417

[144] Pasquier E André N Street J Chougule a Rekhi B Ghosh J. Effective Management of Advanced Angiosarcoma by the Synergistic Combination of Propranolol and Vinblastine-based Metronomic Chemotherapy: A Bench to Bedside Study. EBioMedicine (2016) 6:87–95. 10.1016/J.Ebiom.2016.02.026 27211551 PMC4856748

[145] Banavali s Pasquier E Andre N. Targeted Therapy With Propranolol and Metronomic Chemotherapy Combination: Sustained Complete Response of a Relapsing Metastatic Angiosarcoma. Ecancermedicalscience (2015) 9. 10.3332/Ecancer.2015.499 PMC4303616 25624880

[146] Chow W Amaya Cn Rains s Chow M Dickerson Eb Bryan B. Growth Attenuation of Cutaneous Angiosarcoma With Propranolol-Mediated β-Blockade. JAMA Dermatol (2015) 151(11):1226–9. 10.1001/Jamadermatol.2015.2554 26375166

[147] Barron AJ Zaman N Cole GD Wensel R Okonko DO Francis DP. Systematic Review of Genuine Versus Spurious Side-Effects of Beta-Blockers in Heart Failure Using Placebo Control: Recommendations for Patient Information. Int J Cardiol (2013) 168(4):3572–9. 10.1016/j.ijcard.2013.05.068 PMC3819624 23796325

[148] Wiysonge CS Bradley HA Volmink J Mayosi BM Opie LH. Beta-Blockers for Hypertension. Cochrane Database Syst Rev (2017) 1(1):CD002003. 10.1002/14651858.CD002003.pub5 28107561 PMC5369873

[149] Nam G. T-Type Calcium Channel Blockers: A Patent Review (2012–2018). Expert Opin Ther Pat (2018) 28(12):883–901. 10.1080/13543776.2018.1541982 30372652

[150] Ji Y Chen S. Antihypertensive Medications and Breast Cancer Risk. JAMA Intern Med (2014) 174(4):640. 10.1001/jamainternmed.2013.13749 24711186

[151] Yoshida J Ishibashi T Nishio M. Antitumor Effects of Amlodipine, a Ca2+ Channel Blocker, on Human Epidermoid Carcinoma A431 Cells In Vitro and In Vivo. Eur J Pharmacol (2004) 492(2–3):103–12. 10.1016/j.ejphar.2004.04.006 15178352

[152] Sela S Husain SR Pearson JW Longo DL Rahman A. Reversal of Multidrug Resistance in Human Colon Cancer Cells Expressing the Human MDR1 Gene by Liposomes in Combination With Monoclonal Antibody or Verapamil. J Natl Cancer Inst (1995) 87(2):123–8. 10.1093/jnci/87.2.123 7707383

[153] Zhao L Zhao Y Schwarz B Mysliwietz J Hartig R Camaj P. Verapamil Inhibits Tumor Progression of Chemotherapyresistant Pancreatic Cancer Side Population Cells. Int J Oncol (2016) 49(1):99–110. 10.3892/ijo.2016.3512 27177126 PMC4902079

[154] Ambudkar SV Kim IW Sauna ZE. The Power of the Pump: Mechanisms of Action of P-glycoprotein (Abcb1). Eur J Pharm Sci (2006) 27(5):392–400. 10.1016/j.ejps.2005.10.010 16352426

[155] Ledwitch KV Gibbs ME Barnes RW Roberts AG. Cooperativity Between Verapamil and ATP Bound to the Efflux Transporter P-Glycoprotein. Biochem Pharmacol (2016) 118:96–108. 10.1016/j.bcp.2016.08.013 27531061 PMC5341695

[156] Muller C Goubin F Ferrandis E Cornil-Scharwtz I Bailly JD Bordier C. Evidence for Transcriptional Control of Human Mdr1 Gene Expression by Verapamil in Multidrug-Resistant Leukemic Cells. Mol Pharmacol (1995) 47(1):51–6. 7838133

[157] Muller C Bailly JD Goubin F Laredo J Jaffrézou JP Bordier C. Verapamil Decreases P-glycoprotein Expression in Multidrug-Resistant Human Leukemic Cell Lines. Int J Cancer (1994) 56(5):749–54. 10.1002/ijc.2910560523 7906257

[158] Huang J Duan Q Fan P Ji C Lv Y Lin X. Clinical Evaluation of Targeted Arterial Infusion of Verapamil in the Interventional Chemotherapy of Primary Hepatocellular Carcinoma. Cell Biochem Biophys (2011) 59(2):127–32. 10.1007/s12013-010-9125-9 PMC3042093 20963512

[159] Deshmukh RR Kim S Elghoul Y Dou QP. P-Glycoprotein Inhibition Sensitizes Human Breast Cancer Cells to Proteasome Inhibitors. J Cell Biochem (2017) 118(5):1239–48. 10.1002/jcb.25783 PMC6858998 27813130

[160] Takada K Kashiwagi S Asano Y Goto W Takahashi K Fujita H. Verification of the Effects of Calcium Channel Blockers on the Immune Microenvironment of Breast Cancer. BMC Cancer (2019) 19(1). 10.1186/s12885-019-5828-5 PMC6591916 31234828

[161] Busby J Mills K Zhang SD Liberante FG Cardwell CR. Postdiagnostic Calcium Channel Blocker Use and Breast Cancer Mortality. Epidemiology (2018) 29(3):407–13. 10.1097/EDE.0000000000000814 29608546

[162] Kim SA Moon H Roh JL Kim SB Choi SH Nam SY. Postdiagnostic Use of β-Blockers and Other Antihypertensive Drugs and the Risk of Recurrence and Mortality in Head and Neck Cancer Patients: An Observational Study of 10,414 Person–Years of Follow-Up. Clin Transl Oncol (2017) 19(7):826–33. 10.1007/s12094-016-1608-8 28093700

[163] Wong MCS Tam WWS Lao XQ Wang HHX Kwan MWM Cheung CSK. The Incidence of Cancer Deaths Among Hypertensive Patients in a Large Chinese Population: A Cohort Study. Int J Cardiol (2015) 179:178–85. 10.1016/j.ijcard.2014.10.028 25464439

[164] Chae YK Dimou A Pierce S Kantarjian H Andreeff M. The Effect of Calcium Channel Blockers on the Outcome of Acute Myeloid Leukemia. Leuk Lymphoma (2014) 55(12):2822–9. 10.3109/10428194.2014.901513 PMC4213321 24628293

[165] Karsy M Hoang N Barth T Burt L Dunson W Gillespie DL. Combined Hydroxyurea and Verapamil in the Clinical Treatment of Refractory Meningioma: Human and Orthotopic Xenograft Studies. World Neurosurg (2016) 86:210–9. 10.1016/j.wneu.2015.09.060 26428319

[166] Tingle SJ. Role of Anti-Stromal Polypharmacy in Increasing Survival After Pancreaticoduodenectomy for Pancreatic Ductal Adenocarcinoma. World J Gastrointest Pathophysiol (2015) 6(4):235. 10.4291/wjgp.v6.i4.235 26600982 PMC4644888

[167] Tingle SJ Severs GR Moir JAG White SA. Calcium Channel Blockers in Pancreatic Cancer: Increased Overall Survival in a Retrospective Cohort Study. Anticancer Drugs (2020) 31(7):737–41. 10.1097/CAD.0000000000000947 32639282

[168] Nakai Y Isayama H Sasaki T Mizuno S Sasahira N Kogure H. Clinical Outcomes of Chemotherapy for Diabetic and Nondiabetic Patients With Pancreatic Cancer: Better Prognosis With Statin Use in Diabetic Patients. Pancreas (2013) 42(2):202–8. 10.1097/MPA.0b013e31825de678 23000889

[169] Milroy R. A Randomised Clinical Study of Verapamil in Addition to Combination Chemotherapy in Small Cell Lung Cancer. West of Scotland Lung Cancer Research Group, and the Aberdeen Oncology Group. Br J Cancer (1993) 68(4):813–8. 10.1038/bjc.1993.433 PMC1968611 8398713

[170] Benson AB3 Trump DL Koeller JM Egorin MI Olman EA Witte RS. Phase I Study of Vinblastine and Verapamil Given by Concurrent Iv Infusion. Cancer Treat Rep (1985) 69(7–8):795–9. 4016789

[171] Frishman WH. Calcium Channel Blockers: Differences Between Subclasses. Am J Cardiovasc Drugs (2007) 7(SUPPL. 1):17–23. 10.2165/00129784-200707010-00002 19845073

[172] Kidoguchi S Sugano N Tokudome G Yokoo T Yano Y Hatake K. New Concept of Onco-Hypertension and Future Perspectives. Hypertension (2020) January):16–27. 10.1161/HYPERTENSIONAHA.120.16044 33222548

[173] Roush GC Kaur R Ernst ME. Diuretics: A Review and Update. J Cardiovasc Pharmacol Ther (2014) 19(1):5–13. 10.1177/1074248413497257 24243991

[174] Bendinelli B Masala G Garamella G Palli D Caini S. Do Thiazide Diuretics Increase the Risk of Skin Cancer? A Critical Review of the Scientific Evidence and Updated Meta-Analysis. Curr Cardiol Rep (2019) 21(9):92. 10.1007/s11886-019-1183-z 31352643

[175] Alhanafy AM Labeeb A Khalil A. The Role of Diuretics in Treatment of Aromatase Inhibitors Induced Musculoskeletal Symptoms in Women With non Metastatic Breast Cancer. Asian Pac J Cancer Prev (2018) 19(12):3525–31. 10.31557/APJCP.2018.19.12.3525 PMC6428535 30583679

[176] Mravec B Horvathova L Hunakova L. Neurobiology of Cancer: The Role of β-Adrenergic Receptor Signaling in Various Tumor Environments. Int J Mol Sci (2020) 21(21):1–24. 10.3390/ijms21217958 PMC7662752 33114769

[177] Wong CH Siah KW Lo AW. Estimation of Clinical Trial Success Rates and Related Parameters. Biostatistics (2019) 20(2):273–86. 10.1093/biostatistics/kxx069 PMC6409418 29394327

[178] Prada-Ramallal G Takkouche B Figueiras A. Bias in Pharmacoepidemiologic Studies Using Secondary Health Care Databases: A Scoping Review. BMC Med Res Methodol (2019) 19(1). 10.1186/s12874-019-0695-y PMC6419460 30871502

[179] Ireson CR Alavijeh MS Palmer AM Fowler ER Jones HJ. The Role of Mouse Tumour Models in the Discovery and Development of Anticancer Drugs. Br J Cancer (2019) 121(2):101–8. 10.1038/s41416-019-0495-5 PMC6738037 31231121

[180] Vivarelli S Candido S Caruso G Falzone L Libra M. Patient-Derived Tumor Organoids for Drug Repositioning in Cancer Care: A Promising Approach in the Era of Tailored Treatment. Cancers (Basel) (2020) 12(12):1–22. 10.3390/cancers12123636 PMC7761978 33291603

[181] Hernandez JJ Pryszlak M Smith L Yanchus C Kurji N Shahani VM. Giving Drugs a Second Chance: Overcoming Regulatory and Financial Hurdles in Repurposing Approved Drugs as Cancer Therapeutics. Front Oncol (2017) 7:273(NOV). 10.3389/fonc.2017.00273 29184849 PMC5694537

